# Engineering the lymph node environment promotes antigen-specific efficacy in type 1 diabetes and islet transplantation

**DOI:** 10.1038/s41467-023-36225-5

**Published:** 2023-02-08

**Authors:** Joshua M. Gammon, Sean T. Carey, Vikas Saxena, Haleigh B. Eppler, Shannon J. Tsai, Christina Paluskievicz, Yanbao Xiong, Lushen Li, Marian Ackun-Farmmer, Lisa H. Tostanoski, Emily A. Gosselin, Alexis A. Yanes, Xiangbin Zeng, Robert S. Oakes, Jonathan S. Bromberg, Christopher M. Jewell

**Affiliations:** 1 Fischell Department of Bioengineering, University of Maryland, College Park, 8278 Paint Branch Drive, College Park, MD 20742 USA; 2 Department of Surgery, University of Maryland Medical School, 22 S. Greene Street, S8B06, Baltimore, MD 21201 USA; 3 Department of Veterans Affairs, VA Maryland Health Care System, 10. N Green Street, Baltimore, MD 21201 USA; 4 Department of Microbiology and Immunology, University of Maryland Medical School, 685 West 30 Baltimore Street, HSF-I Suite 380, Baltimore, MD 21201 USA; 5 Robert E. Fischell Institute for Biomedical Devices, 8278 Paint Branch Drive, College Park, MD 20742 USA; 6 Marlene and Stewart Greenebaum Cancer Center, 22 S. Greene Street, Suite N9E17, Baltimore, 32 MD 21201 USA

**Keywords:** Biomedical engineering, Autoimmunity, Transplant immunology, Biomaterials

## Abstract

Antigen-specific tolerance is a key goal of experimental immunotherapies for autoimmune disease and allograft rejection. This outcome could selectively inhibit detrimental inflammatory immune responses without compromising functional protective immunity. A major challenge facing antigen-specific immunotherapies is ineffective control over immune signal targeting and integration, limiting efficacy and causing systemic non-specific suppression. Here we use intra-lymph node injection of diffusion-limited degradable microparticles that encapsulate self-antigens with the immunomodulatory small molecule, rapamycin. We show this strategy potently inhibits disease during pre-clinical type 1 diabetes and allogenic islet transplantation. Antigen and rapamycin are required for maximal efficacy, and tolerance is accompanied by expansion of antigen-specific regulatory T cells in treated and untreated lymph nodes. The antigen-specific tolerance in type 1 diabetes is systemic but avoids non-specific immune suppression. Further, microparticle treatment results in the development of tolerogenic structural microdomains in lymph nodes. Finally, these local structural and functional changes in lymph nodes promote memory markers among antigen-specific regulatory T cells, and tolerance that is durable. This work supports intra-lymph node injection of tolerogenic microparticles as a powerful platform to promote antigen-dependent efficacy in type 1 diabetes and allogenic islet transplantation.

## Abstract (web summary)

Antigen-specific tolerance represents a promising strategy to treat type 1 diabetes and islet allograft rejection. Here, the authors deliver immune signals to lymph nodes to promote antigen-specific regulatory T cells and prevent disease in models of type 1 diabetes and allogenic islet transplantation.

## Introduction

The adaptive immune response provides antigen-specific protection against foreign pathogens while preserving tolerance against self-tissue. However, tolerance breaks down during autoimmune diseases such as type 1 diabetes (T1D), where pancreatic islets are targeted and destroyed. While T1D can be managed with frequent insulin administration, multiple comorbidities exist including cardiovascular^1^ and gastrointestinal disease^2^. There are currently no approved therapies targeting the underlying immunopathology of T1D.

Existing clinical therapies for autoimmune disease aim to restrain inflammation and self-reactivity using systemic administration of immunosuppressive drugs or modulatory therapies^3^. These treatments are not curative and require life-long compliance. Even the newest monoclonal antibodies do not distinguish between healthy and self-reactive cells, leading to serious side effects and non-specific immunosuppression^4^. For example, a monoclonal antibody targeting CD3—which suppresses and depletes effector T cells^5^—has delayed the loss of insulin production in patients previously diagnosed with T1D. This drug was recently demonstrated as the first immunotherapy in a clinical trial to significantly delay progression in at-risk T1D patients^6,7^. While more specific than classical immunosuppressants, this treatment is not curative and cannot distinguish healthy and autoreactive T cells. Another therapeutic approach for T1D is the transplantation of allogenic islets to restore control of insulin. However, these grafts are allogeneic, resulting in an attack by the recipient (i.e. host) T and B lymphocytes^8,9^. This requires nonspecific systemic immunosuppression to ameliorate graft rejection^10^. Novel treatments with molecular specificity are needed to address these persistent unmet clinical challenges.

An experimental therapeutic concept in autoimmune disease is the induction of tolerance against specific self-antigens, without inhibiting normal adaptive immune responses needed to fight infection^11,12^. This strategy involves the administration of vaccine-like treatments that co-deliver relevant autoantigens with regulatory immune cues to redirect autoantigen-specific T cells toward populations with regulatory immune functions^13–20^. A number of these approaches focus on generation of regulatory T cells (T_REG_), which exhibit potent tolerizing capabilities^21^. Because the fate of T cells during antigen encounter depends on signal integration in lymph nodes (LNs)—tissues that coordinate immunity^22^—a challenge of this approach is ensuring self and regulatory cues are presented in appropriate combinations and concentrations in LNs. Recently, intra-lymph node (*iLN)* injection of soluble vaccines have shown promise in allergy^23,24^, cancer^25,26^, and T1D^27,28^. In these therapeutic contexts, *i.LN* injection has demonstrated striking potency and dramatic dose sparing in early trials^25^. While these trials utilized soluble signals which quickly drain from LNs, we’ve developed a system which employs *i.LN* injection of immune signals encapsulated in microparticle (MP) depots which are synthesized to be diffusion-limited and too large to drain out of LNs^29^. Instead, the MPs slowly degrade, locally concentrating immune cues that condition the local LN environment to direct immune cell fate.

We have previously applied our modular *i.LN* platform to directly deposit antigens and modulatory immune cues, such as rapamycin (Rapa)—an inhibitor of the mammalian target of rapamycin (mTOR) pathway—in LNs to combat immune-mediated neurodegeneration during multiple sclerosis^30^. This work revealed *i.LN* treatment promoted tolerance in tandem with an increase in polyclonal T_REG._ However the effect of *i.LN* treatment on antigen-specific response has not been explored mechanistically. There are key questions that remain unknown with this platform, including the distinct roles of antigen and Rapa towards promoting a durable tolerogenic T cell response, how these immune signals affect structure of treated and untreated LNs is unknown, and lastly, whether this approach promotes tolerance or suppression in models of autoimmunity or alloimmune response driven by T cell responses against multiple antigens. To answer these questions, here we leverage our *i.LN* platform to directly study how immune cues impact the local LN microenvironment during antigen-specific autoimmunity in treated and untreated nodes in autoimmune and alloimmune disease models. We hypothesized application of this LN targeted approach to T1D and islet transplantation could obviate the need to administer high systemic doses of immunosuppressants through localizing tolerizing signals in LNs for tunable times and concentrations.

**Fig. 1:**
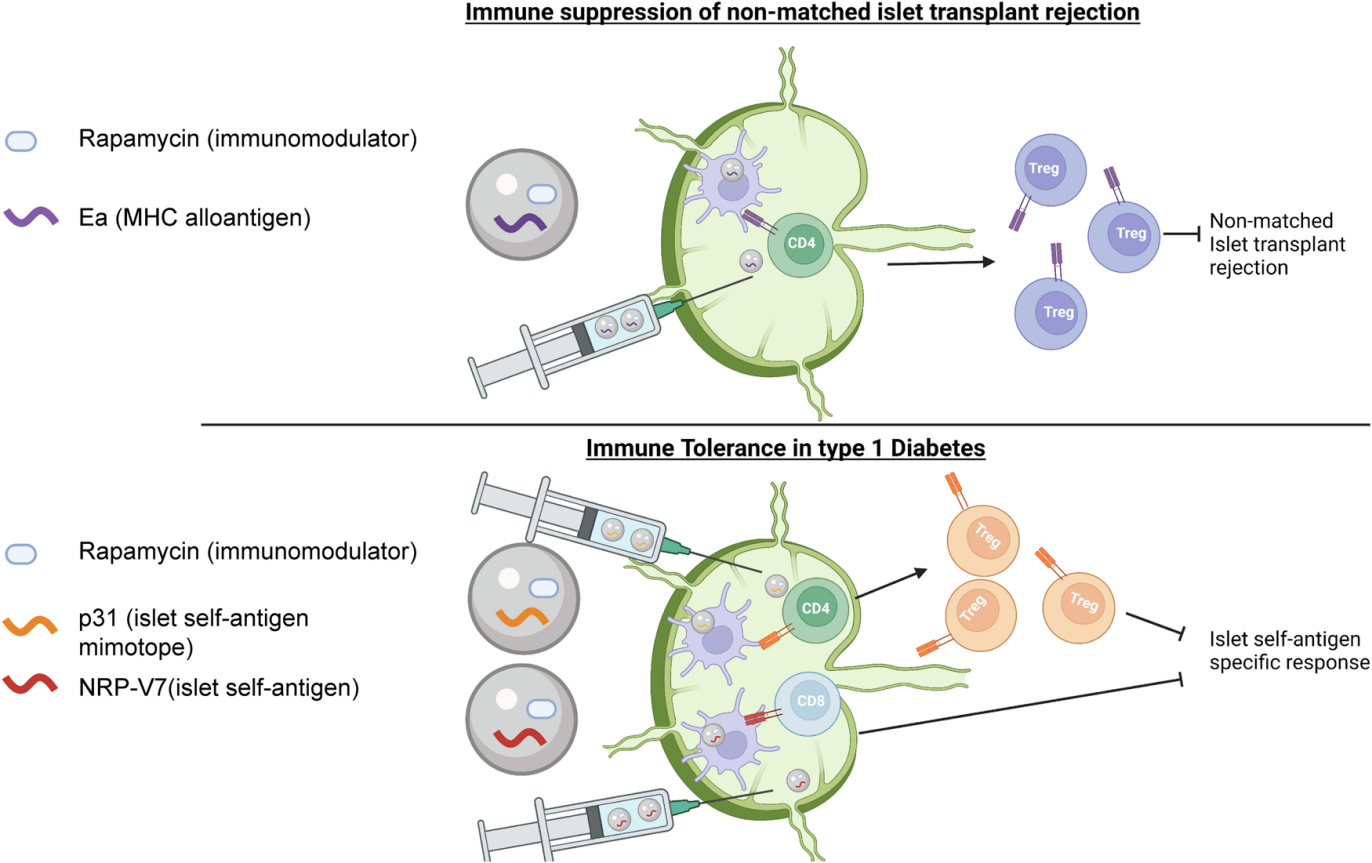
Overview schematic of *iLN* microparticle depot strategy to promote tolerance in T1D and allogenic islet transplantation. Intra-lymph node (*iLN*). This figure was created with Biorender.com.

Here we have applied this system to two distinct T1D contexts: pre-clinical models of CD4 and CD8-mediated T1D, and an allogenic islet transplant model in which full MHC mismatched donor islets are transferred to recipients after islet depletion. We synthesized MPs co-encapsulating Rapa and three distinct peptide antigens targeted in T1D or allograft rejection. In both models, MP formulations with appropriate antigens effectively prevented disease, and co-encapsulation of self-antigen with Rapa was critical for optimal protection in each case. MP treatment expanded antigen-specific T_REG_ in treated and untreated LNs, and resulted in structural reorganization of LNs with microdomains associated with induction of tolerance. Finally, antigen-specific T_REG_ displayed markers of enhanced persistence, and a single MP treatment stopped disease progression even when disease was induced at an extended timepoint following treatment. In contrast to all existing therapies which are administered systemically, local LN engineering could provide selective suppression or tolerance for autoimmune and alloimmune diseases without broad exposure to potent immunosuppressants (Fig. 1).

## Results and discussion

### Tolerogenic MPs promote antigen-specific regulatory responses across multiple T1D-relevant self- and allo-antigens

We hypothesized *iLN* depots could promote antigen-specific tolerance against pancreatic autoantigens and donor allo-antigens attacked on islets. Because in the NOD model of T1D tolerance can span multiple epitopes, we further hypothesized our platform would enable different antigens to be used interchangeably to drive tolerance across key epitopes underpinning T1D pathology. To test this, we selected NOD models of T1D where T1D can be induced by transfer of islet reactive T cells. Transgenic T cell receptor (TCR) mice with a NOD background have been engineered to express CD8 or CD4 T cells that are reactive to islet auto-antigens, and can be activated by mimotopes of islet peptide antigens. NOD8.3 TCR transgenic mice express CD8 TCRs which can be activated by the NRP-V7^31–33^ mimotope, and BDC2.5 transgenic mice express CD4 T cells that can be activated by the p31^34–36^ mimotope. For islet transplantation, we selected a full MHC mismatched islet transplant model using only one of the many I-E^d^ class II MHC (EA) disparities to redirect the allogeneic response from immunity to suppression (Fig. 1).

**Fig. 2:**
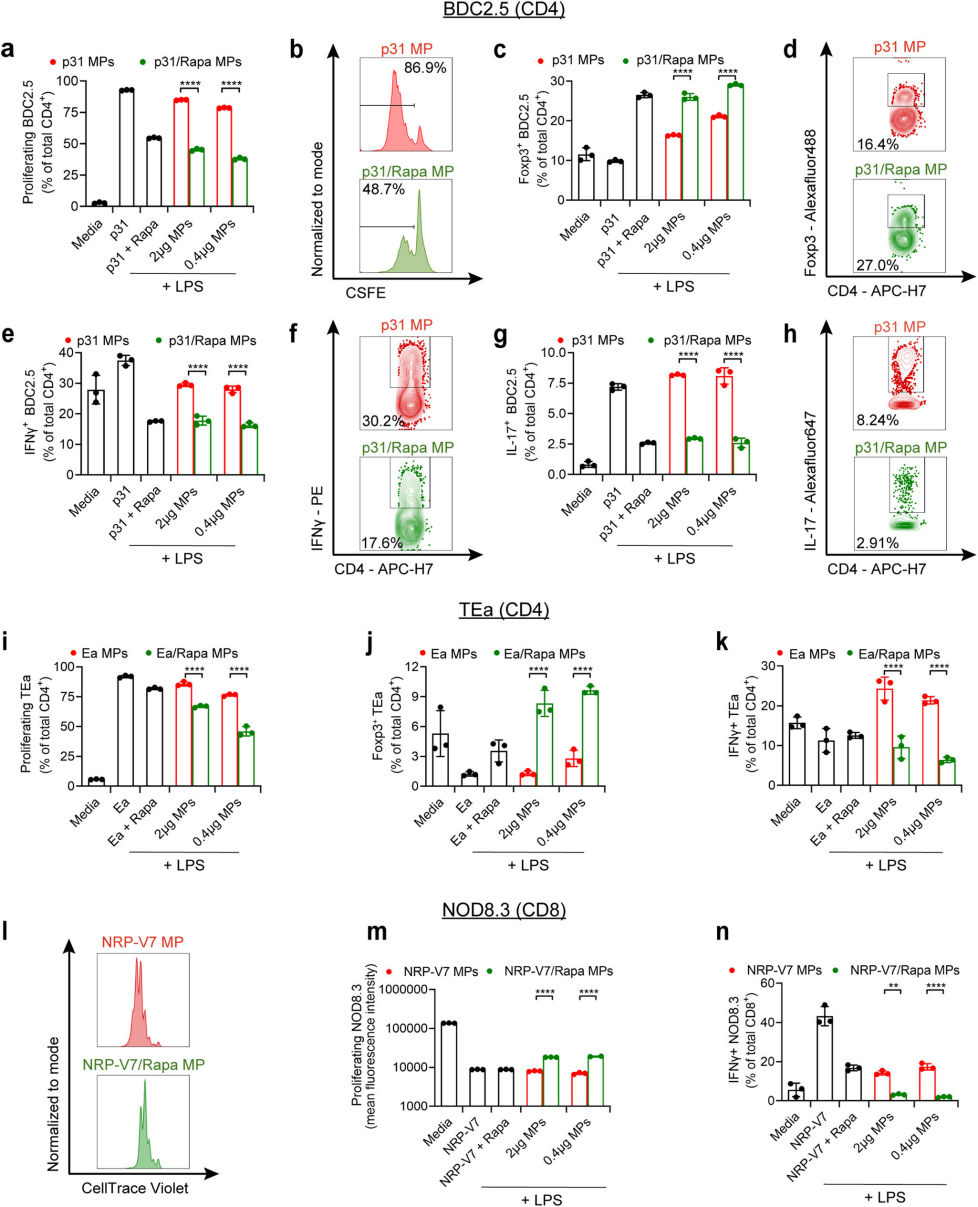
MPs encapsulating relevant antigens and Rapa promote suppressive T cell responses in vitro. BDC2.5 T cell co-cultures were treated with p31 MP or p31/Rapa MPs. **a** Quantification of BDC2.5 T cell proliferation and **b** representative flow cytometry plots of CSFE dilution are shown. **c** Quantification of Foxp3 expression and **d** representative flow cytometry plots are shown. **e** Quantification of IFNγ expression and **f** representative flow cytometry plots are shown. **g** Quantification of IL-17 expression and **h** representative flow cytometry plots are shown. TEa T cell co-cultures were treated with Ea MP or Ea/Rapa MPs. Quantification of **i** proliferation, **j** Foxp3 expression and **k** IFNγ expression are shown. NOD8.3 T cell co-cultures were treated with NRP-V7 MP or NRP-V7/Rapa MPs. **l** Representative flow cytometry plots showing proliferation and quantification of **m** proliferation and **n** IFNγ production are shown. *N* = 3 individual culture wells for all experiments. Plots represent mean ± s.d. One-way ANOVA with Tukey’s post test used to compare treatment groups and comparisons between matched doses of p31 and p31/Rapa MPs are shown. *p < 0.05, **p < 0.01, ***p < 0.001, ****p < 0.0001. Source data are provided as a Source Data file.

Poly(glycolide-co-lactide) (PLGA) MP depots were synthesized co-encapsulating Rapa with each self- (i.e., NRP-V7, p31) or allo-antigen (i.e., Ea) (Fig. 1). MP properties for each formulation are listed in Supplementary Table 1. In co-cultures consisting of DCs from NOD mice and T cell receptor transgenic CD4 T cells from BDC2.5 transgenic mice that recognize p31 antigen presented by DCs, p31/Rapa MP partially inhibited T cell proliferation compared to p31 (Fig. 2a, b). However, phenotypic analysis of the expanding BDC2.5 T cells revealed p31/Rapa MP significantly increased T_REG_ frequency (Fig. 2c, d, Supplementary Fig. 1a). This polarization was also associated with decreased production of T_H_1- and T_H_17-associated inflammatory-cytokines IFNγ (Fig. 2e, f) and IL-17 (Fig. 2g, h), respectively. Analogous shifts from inflammation to tolerance (i.e., T_REG_) were observed during co-culture of DCs with TEa transgenic CD4 T cells treated with Ea/Rapa MPs, but not Ea MPs (Fig. 2i–k). Since almost no endogenous T_REG_ are present in TEa mice, Ea/Rapa MPs promote a TEa T_REG_ response through conversion of non-T_REG_ (Supplemental Fig. 2a, b). Lastly, during co-culture of DCs and NOD8.3 CD8 T cells, NRP-V7/Rapa MP significantly restrained proliferation (Fig. 2l) and IFNγ production (Fig. 2m, n, Supplementary Fig. 1b). Together these data demonstrate antigen-specific tolerization and control of inflammation in three distinct T1D contexts that span self- and alloantigen, as well as CD4- (i.e., BDC2.5, TEa) and CD8-driven (i.e., NOD8.3) inflammatory T cells.

### Direct LN injection of tolerogenic MPs promotes tolerance in pre-clinical models of T1D and islet transplantation

**Fig. 3:**
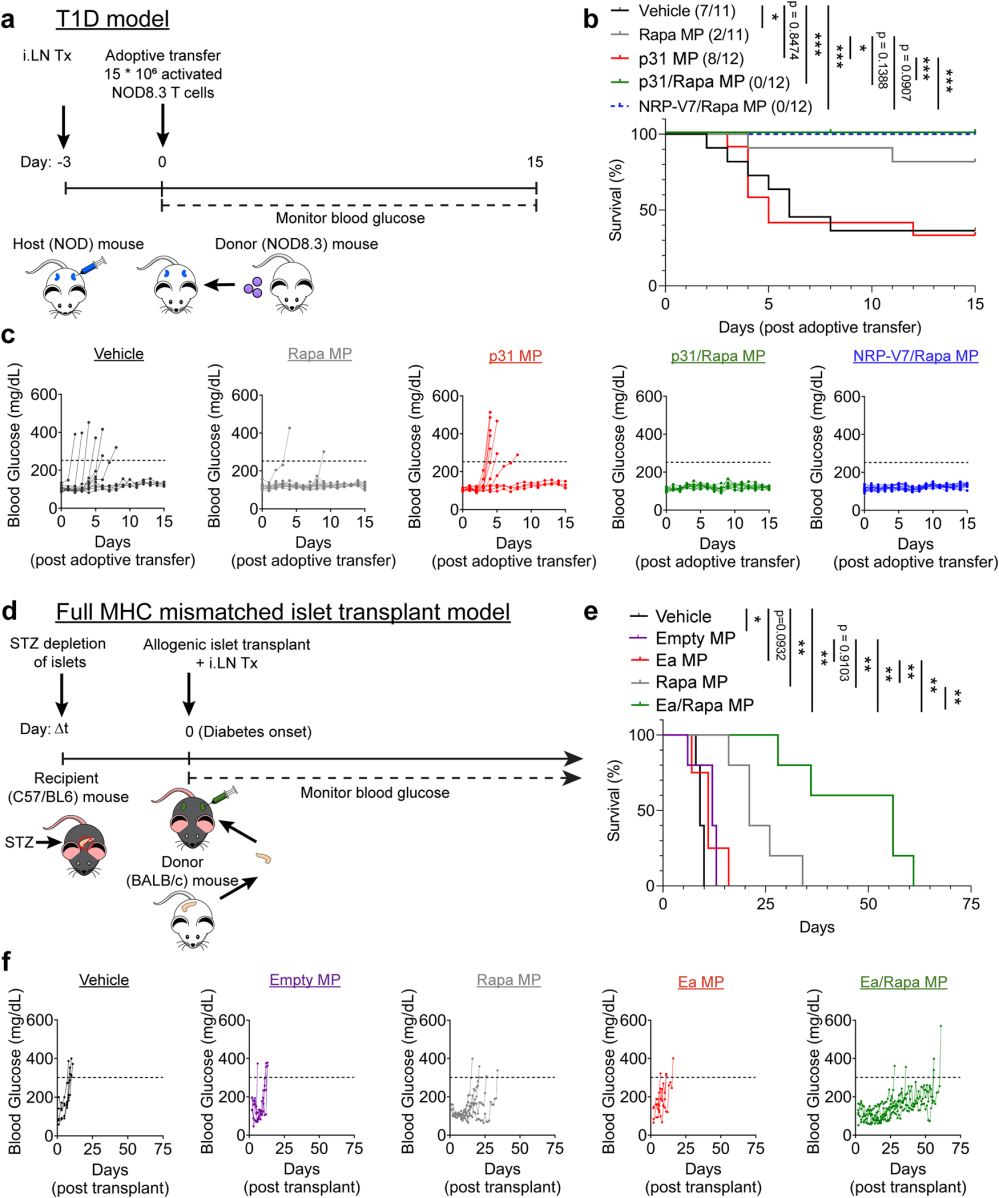
*iLN* injection of tolerogenic MPs promotes tolerance in T1D and suppression of graft rejection in allogenic islet transplantation. **a** Schematic representation of prophylactic *iLN* treatment in the NOD8.3 model of T1D. **b** Survival curve with number of mice which developed disease out of the total mice per group indicated in the parenthesis in the legend. **c** Individual blood glucose traces for mice in each group from **b**. **d** Schematic representation of *iLN* treatment in the non-matched islet transplant model. **e** Survival curve for experiment detailed in **d**. **f** Individual blood glucose traces for mice in each group (**d**). *N* = 5 mice for all groups except *N* = 4 mice for Rapa MP treatment group. Two-tailed log-rank (Mantel-Cox) was used for all pairwise comparisons between each treatment for survival curves in **b**. *p < 0.05, **p < 0.01, ***p < 0.001, ****p < 0.0001. Source data are provided as a Source Data file. Intra-lymph node (*iLN*), streptozotocin (STZ).

As an initial setting to test the tolerizing ability of MPs in T1D, we used an accelerated model of T1D where ex vivo activated NOD8.3 T cells are adoptively transferred into NOD mice (Fig. 3a). Mice were treated *iLN* (Supplemental Fig. 3) 3 days prior to T cell transfer to test if NPR-V7/Rapa MPs could inhibit T1D, and importantly, if MPs loaded with a different disease-relevant peptide (i.e., p31/Rapa MPs) could also prevent disease. Excitingly, a single treatment of either NRP-V7/Rapa MP or p31/Rapa MPs prevented disease in all animals, whereas p31-only MPs provided no benefit relative to the vehicle (Fig. 3b, c). These data indicate the inclusion of either a CD4-dominant (p31) or CD8-dominant (NRP-V7) epitope provided efficacy, even when the disease was mediated by CD8 reactivity (i.e., NOD8.3 TCR transgenic T cells). We also note Rapa MPs partially inhibited disease, which may have resulted from nonspecific transient suppression. To benchmark *iLN* MPs against a conventional treatment formulation and route, mice were treated *iLN* with p31/Rapa MPs or *i.p*. with free p31 and Rapa 3 days before adoptive transfer of NOD8.3 T cells (Supplemental Fig. 4a). *iLN* provided significant efficacy compared to *i.p* treatment of free p31 and Rapa (Supplemental Fig. 4b, c). In studies discussed later, we employed additional regimens to further investigate the role of each component and the durability of induced tolerance. Future studies will focus on testing the ability of *iLN* p31/Rapa MPs to inhibit disease in the spontaneous NOD model of T1D.

We next tested the ability of *iLN* MPs to inhibit islet allograft rejection in a full MHC mismatched transplantation model. Islets were chemically depleted in C57BL/6 mice using streptazotocin (STZ), followed by transplantation of allogeneic islets from BALB/c mice underneath the kidney capsule (Fig. 3d). In this model, alloantigen of donor islets is targeted by the host adaptive immune response. *iLN* treatment was administered once on the first day of the disease, determined by hyperglycemia. For peptide antigen to induce suppression, we chose only a single I-E^d^ α-chain disparity between donor and recipients, leaving untargeted a multitude of other MHC class I and minor antigen differences between the two mouse strains. Vehicle, Empty MP, and Ea MP treated cohorts all rapidly rejected transplanted islets (Fig. 3e, f), with median survivals of 9, 12 and 11 days, respectively. Similar to the NOD8.3 model, Rapa MP alone modestly prolonged survival compared to Vehicle or MPs encapsulating Ea antigen alone. Intriguingly, Ea/Rapa MPs provided significant therapeutic benefit with a median survival of 56 days vs 21 days and 9 days for Rapa MPs and vehicle, respectively (Fig. 3e, f). While Ea/Rapa MPs dramatically prolonged survival in the islet transplantation model, they were unable to fully restore tolerance unlike p31/Rapa MPs or NRP-V7/Rapa MPs in the T1D model. This difference in efficacy may be due to the much stronger immune response induced against the multitude of alloantigens targeted in the full MHC mismatched transplantation model. Future experiments will investigate if co-delivering multiple alloantigens with Rapa can more effectively suppress this broad alloimmune response. This approach could offer broader, personalized-like tolerance, or dose sparing. Together, efficacy in these pre-clinical models reveals depots potently combat T1D and islet rejection with one treatment, and co-encapsulation of Rapa and disease-relevant—but not necessarily disease-induction—epitope maximize efficacy.

### p31/Rapa MPs prime antigen-specific T cells in treated and untreated LNs while suppressing mTOR activity

**Fig. 4:**
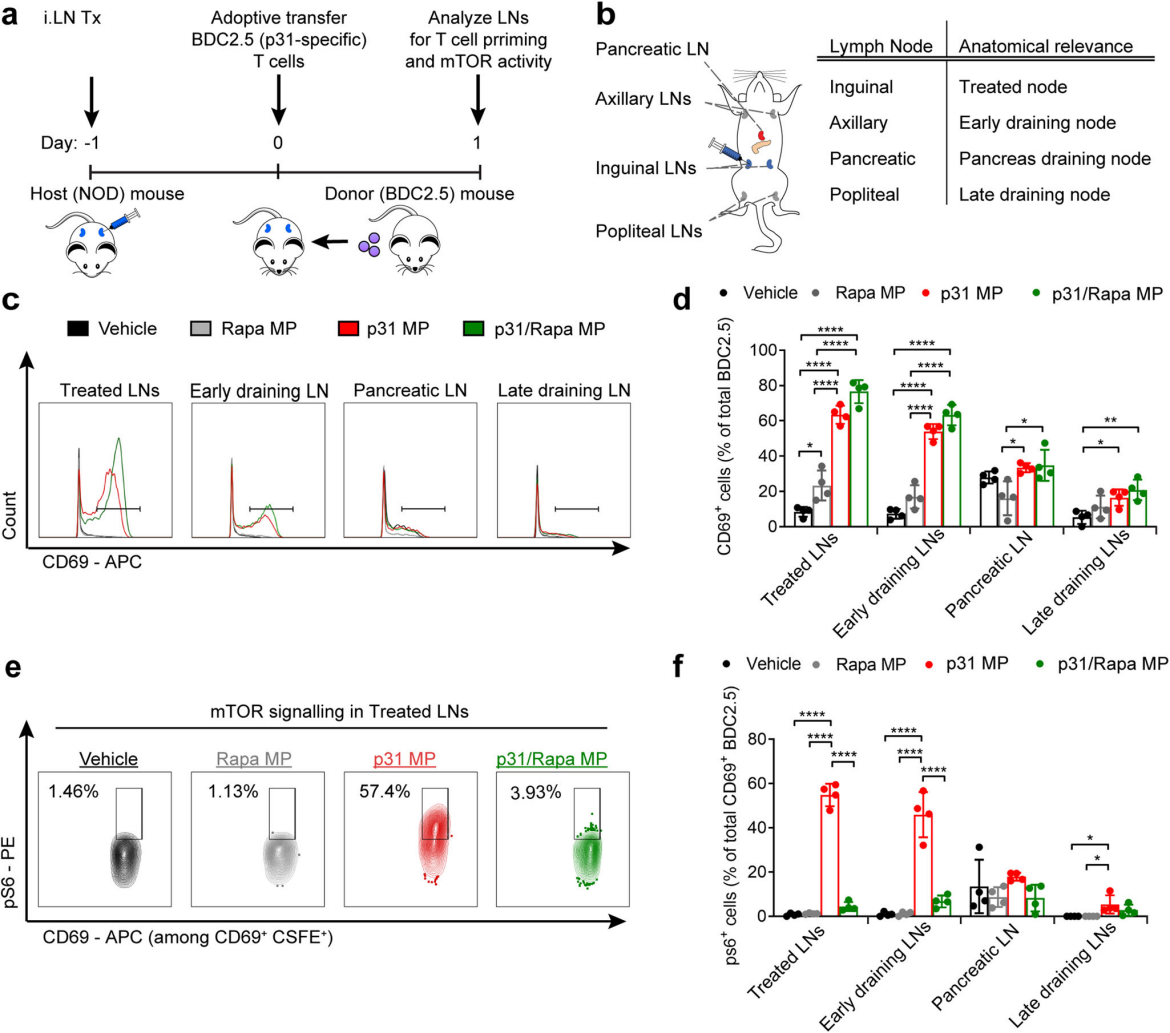
p31/Rapa MPs promote antigen-specific T cell priming while inhibiting mTOR in vivo. **a** Schematic representation of MP treatment and experimental readout for BDC2.5 T cell adoptive transfer experiment. **b** Illustration of lymph nodes collected and their anatomical relevance to treatment. **c** Representative flow cytometry traces and **d** quantification of CD69 expression among p31 specific T cells. **e** Representative flow cytometry traces of phosphorylated s6 among CD69^+^ p31 specific T cells in treated LNs and **f** quantification in all LNs. *N* = 4 mice for all experiments, and paired LNs (i.e. inguinals, axillary, popliteal) were pooled for each mouse. The mean ±; s.d. is shown. One way ANOVA with Tukey’s post hoc test was used to compare each treatment within individual lymph nodes. *p < 0.05, **p < 0.01, ***p < 0.001, ****p < 0.0001. Source data are provided as a Source Data file. Intra-lymph node (*iLN*), phosphorylated ribosomal s6 protein (ps6).

Since MP treatment promotes tolerance with respect to disease pathology, we next investigated the mechanism and localization of T cell polarization by testing if MPs prime antigen-specific T cells and inhibit mTOR in treated and untreated LNs. NOD mice were injected with p31/Rapa MPs in the inguinal LNs, then BDC2.5 T cells were adoptively transferred to ensure a large population of antigen-specific T cells for analysis (Fig. 4a). BDC2.5 T cells were labeled with CSFE prior to cell transfer to allow tracking at early timepoints. One day after transfer we isolated the transferred cells from LNs (Fig. 4b) and analyzed them for CD69 and phosphorylated ribosomal s6 protein (ps6) expression. CD69 is an early activation marker upregulated within hours of antigen-specific stimulation, while ps6 correlates with increased mTOR signaling. BDC2.5 T cells in treated LNs of mice receiving MP formulations containing p31 antigen (i.e., p31 with or without Rapa) displayed similar levels of CD69 expression, which were elevated compared to cells in treated LNs of mice receiving MP formulations without antigen (Fig. 4c, d, Supplementary Fig. 5a). Interestingly, this trend was also observed in the untreated axillary LNs, which drain the treated inguinal LNs at early timepoints, and to a lesser extent in the pancreas-draining LNs and the distant later-draining popliteal LNs (Fig. 4c, d). These data suggest the systemic presentation of either the p31 peptide or endogenous autoantigen. Concurrently with the upregulation of early activation markers, we discovered inclusion of Rapa in MPs dramatically suppressed phosphorylation of s6 through the mTOR pathway within these cells (Fig. 4e, f, Supplementary Fig. 5b). Once again, this trend was present in treated and untreated LNs. Together these data demonstrate p31/Rapa MPs prime antigen-specific T cells and modulate mTOR activity, a combination of features that we hypothesized could provide sufficient stimulus for T cell expansion, while polarizing these self-specific cells toward T_REG_.

### MPs expand antigen-specific T_REG_ in treated and untreated LNs

**Fig. 5:**
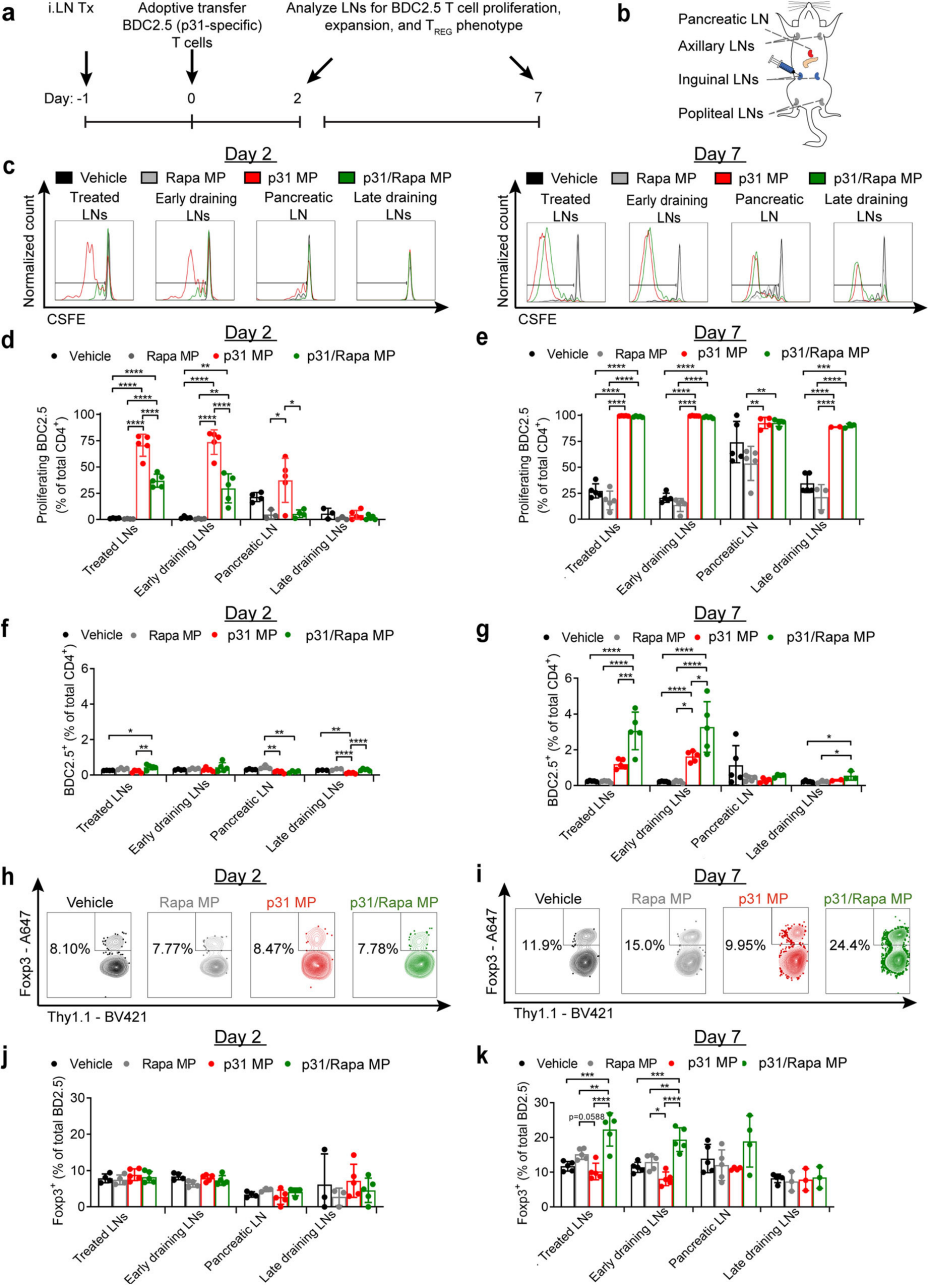
p31/Rapa MPs expand antigen-specific T_REG_ in treated and untreated LNs. **a** Schematic representation of MP treatment and experimental readout for BDC2.5 T cell adoptive transfer experiment. **b** Illustration of LNs collected and analyzed. **c** Representative flow cytometry traces and quantification of p31 specific T cell proliferation at **d** day 2 and **e** day 7. Quantification of p31 specific T cell frequency at **f** day 2 and **g** day 7. Representative flow cytometry traces showing Foxp3 expression among p31 specific T cells in treated LNs at **h** day 2 and **i** day 7. Quantification of Foxp3 expression among p31 specific T cells at **j** day 2 and **k** day 7 in each LN analyzed. *N* = 5 mice for all groups in all experiments, and paired LNs were pooled for each mouse. The mean ±  s.d. is shown. For all comparisons in this figure between mice treated with different treatments, One-way ANOVA with Tukey’s post hoc test was done between all treatment groups within each individual LN. All comparisons between the Rapa MP and p31/Rapa MP treatment groups among different tissues were also done using One way ANOVA with Tukey’s post hoc test, and the *p* values from this analysis are listed in Supplemental Table 2. *p < 0.05, **p < 0.01, ***p < 0.001, ****p < 0.0001. Source data are provided as a Source Data file. Intra-lymph node (*iLN*).

To investigate further the above-mentioned hypothesis, we tested if MP treatment expands antigen-specific T_REG_ by again treating NOD mice *iLN* with MPs and transferring CSFE labeled BDC2.5 T cells (Fig. 5a). Two and seven days after T cell transfer, treated and non-treated LNs were isolated (Fig. 5b). In this experiment T cells were from BDC2.5 mice with a Thy1.1 syngeneic marker. This was used to enable tracking at later timepoints when CSFE dilution due to proliferation makes it overwise impossible to distinguish transferred and host cells. At 2 days post transfer—while both p31 MPs and p31/Rapa MPs induced BDC2.5 T cell proliferation (Fig. 5c, d, Supplementary Fig. 6), the frequency of proliferated cells was significantly higher in the treated, early-draining, and pancreatic LNs (untreated) of mice treated with p31 MP relative to p31/Rapa MP treatment (Fig. 5d). Seven days post transfer, nearly all BDC2.5 T cells in mice treated with p31 or p31/Rapa MPs had proliferated in each LN examined, including distant, later-draining LNs (Fig. 5c, e). The total degree of proliferation was higher among T cells in mice treated with p31 MPs compared to p31/Rapa MPs in treated LN (Supplementary Fig. 7a–c). However, in each case the frequency of proliferated T cells was significantly higher in p31 MP or p31/Rapa MP-treated mice compared to vehicle or Rapa MP-treated mice in each LN. At day 2, the total frequency of BDC2.5 T cells in LNs among all CD4 T cells was low (<0.5% for each group in each LN) (Fig. 5f). By day 7, the frequency of BDC2.5 T cells increased in both treated and draining LNs of mice receiving p31/Rapa MPs and p31 MPs, but intriguingly, p31/Rapa MPs caused significantly greater increases than p31 MPs, representing a threefold expansion in treated LNs. (Fig. 5g). Finally, while analysis of Foxp3 expression showed no differences among BDC2.5 T cells at day 2 in any LN (Fig. 5h, j), by day 7, p31/Rapa MP treatment dramatically expanded T_REG_ frequencies and numbers (Supplementary Fig. 7f) among antigen-specific T cells in treated and the early-draining LNs compared to all treatment groups (Fig. 5i, k, Supplementary Table 2). In addition to T_REG_, p31-specific Foxp3^-^ T cells (non-T_REG_) also proliferated in mice treated with p31/Rapa MPs (Supplementary Fig. 7a–d). p31/Rapa MP treatment also increased T_REG_ frequency among the total CD4 population compared to p31 MPs (Supplementary Fig. 7e). Since BDC2.5 mice develop endogenous T_REG_^37^, the increased T_REG_ frequency among transferred cells may be driven by p31/Rapa MP-induced maintenance or expansion of pre-existing natural or peripheral T_REG_, de novo generation of T_REG_, or conversation of conventional T cells. Future studies will explore these possibilities. Together these data demonstrate the presence of cognate antigen is required to expand antigen-specific T cells in treated and untreated LNs, and inclusion of Rapa is necessary to polarize antigen-specific T cells to T_REG_.

The release rate of antigen and Rapa may influence tolerance induction through modulating the dose and persistence of antigen and Rapa in LNs during T cell priming, thus impacting the magnitude and phenotype of the T cell response^38^. In vitro release studies demonstrated rapamycin and p31 were released over time from MPs, with p31 displaying more rapid release. 88 ± 5.1% of total encapsulated p31 was released and 18.4 ± 8% of total Rapa were released after 48 h (Supplementary Fig. 8). While MP formulations may be tuned in future studies to determine an optimum for promoting tolerance, in this study we utilized MPs formulated similarly to MPs employed in our previous studies where tolerance was achieved in mouse models of multiple sclerosis^30^. We found p31-specific T cells proliferated in injected LNs of mice treated with p31/Rapa MPs 3 weeks after MP injection, demonstrating prolonged antigen release and presentation following MP injection in vivo (Supplementary Fig. 9a, b).

Previous work in multiple models of autoimmune disease has demonstrated systemic delivery of antigen in particulate form in the absence of a regulatory cue can promote antigen-specific tolerance^12,18,39–43^. The mechanisms involved in these studies generally leverage antigen processing through non-inflammatory pathways by antigen-presenting cells (APCs) in LNs, spleens, and liver to restrain antigen-specific immune response. Presentation of antigen in these contexts results in functional deletion or exhaustion of auto-antigen-specific effector T cells and promotion of active tolerance through the generation of T_REG_. In contrast, *iLN* injection of MPs delivering antigen alone expands antigen-specific T cells, does not increase antigen-specific T_REG_ (Fig. 5k) and does not inhibit disease in T1D (Fig. 3b) or islet transplantation (Fig. 3e). These data suggest mechanistically self-antigen is not processed in a tolerogenic manner when delivered *iLN* alone, and instead provides necessary activation and expansion of antigen-specific T cells which are then polarized by Rapa to drive regulatory phenotype. Interestingly, *iLN* treatment with p31/Rapa MPs does not exhaust antigen-specific T cells but promotes active tolerance which may provide durable control of autoimmune reactions. Further study is needed to elucidate the exact role antigen-specific T_REG_ play in promoting tolerance. Future studies will also investigate the interplay of expanded T_REG_ and inflammatory T cell phenotypes such as T_H_1 or T_H_17. While the expanded non-T_REG_ cells may have the potential for pro-inflammatory response, it is clear they are controlled in vivo, and the overall antigen-specific response is tolerogenic (Fig. 3b, e).

### Antigen delivered in MPs is presented in treated and untreated LNs, with presentation in untreated LNs independent of APC migration

**Fig. 6:**
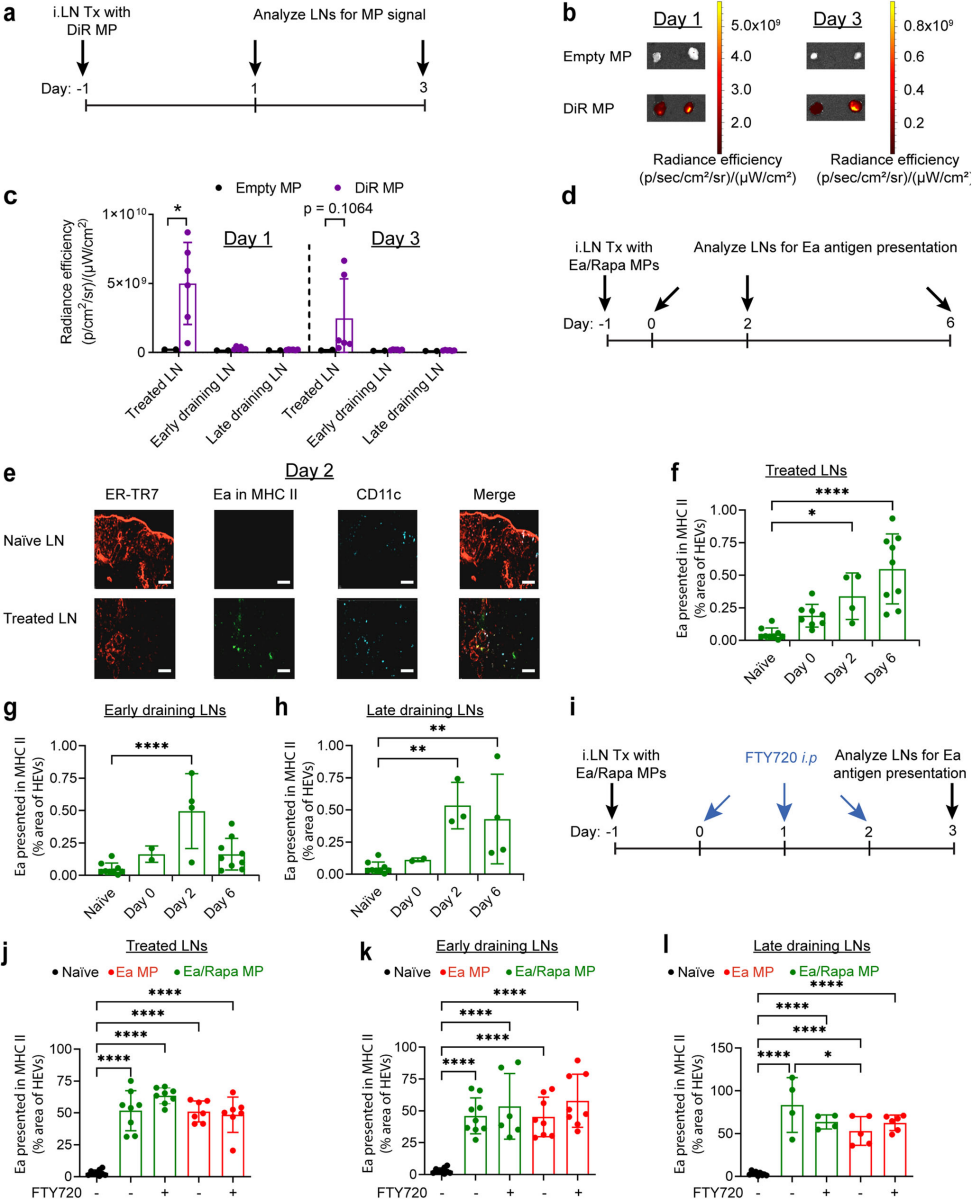
Ea is presented in treated and untreated LNs after Ea/Rapa MP treatment. **a** Schematic representation of experiment in panels **b**–**d**. **b** IVIS images of excised treated LNs. **c** Quantitation of DiR MP signal in excised LNs. Two-tailed Welch’s *t* test was used to compare both treatment groups within individual lymph nodes at each time point. Individual LNs were used as single data points. Mean ± s.d. is shown. *N* = 3 mice for DiR MP treatment groups and *N* = 1 mouse for empty MP treatment group at each timepoint. Each LN is represented as an individual data point. **d** Schematic representation of experiment in panels **f**–**i**. **e** Representative immunofluorescent images of LNs stained with ER-TR7, CD11c, YAe; Scale bar = 50 μm. Quantification of Ea presentation in MHC-II (YAe) in **f** treated, **g** early-draining and **h** late-draining LNs. *N* = 2 mice for each timepoint, with 2 or 3 sections per LN. One way ANOVA with Dunnett’s post hoc test was used to compare percent YAe area to the naive control at each timepoint. **i** Schematic representation of experiment in panels **k**–**m**. Quantification of Ea presentation in MHC-II in **j** treated, **k** early-draining and **l** late-draining LNs after FTY720 treatment. One-way ANOVA with Tukey’s post hoc test was used to compare each group. *N* = 2 mice for each timepoint, with 2 or 3 sections per LN. Mean ± s.d. is shown for each sample in each experiment. *p < 0.05, **p < 0.01, ***p < 0.001, ****p < 0.0001. Source data are provided as a Source Data file. Intra-lymph node (*iLN*).

The finding that after LN injection of MPs containing antigen, CD69—which is transiently upregulated upon stimulation and facilitates temporary retention in LNs^44^—is upregulated in both treated and non-treated LNs (including non-pancreas draining LNs) suggests antigen delivered in MPs is presented in these sites. Since these MPs are size restricted and do not drain through efferent lymphatics, there are two likely mechanisms for delivery to LNs: drainage through lymphatics after release from MPs or trafficking by APCs after internalization of MPs. To confirm MPs are retained in treated LNs, mice were injected *iLN* with unlabeled MPs, or MPs encapsulating a fluorescent dye, DiR (DiR MPs) (Fig. 6a). 2 and 4 days after MP treatment, treated, early-draining and pancreatic-draining LNs were isolated and imaged by IVIS for DiR signal. No DiR signal was detected in any LNs receiving unlabeled MP formulations. However, there was significantly increased DiR signal in LNs treated with DiR MPs 2 days after treatment. In these groups DiR signal was only detectable in treated LNs, supporting local MP retention in treated LNs (Fig. 6b, c). These data demonstrate the restriction of large cargo reservoirs to the treated LNs using intra-LN depots.

To confirm antigen presentation in untreated LNs, we directly investigated the trafficking and presentation of antigen from MPs in untreated LNs using the Y-Ae mAb which directly recognizes the EA peptide when presented in by MHC class II of the recipients^45^. To study Ea presentation, mice were immunized *iLN* with Ea/Rapa MPs on day −1, then the treated and untreated LNs were stained for Ea presentation in areas of LNs surrounding high endothelial venules (HEVs) on days 0, 2, and 6 (Fig. 6d) in the cortical ridge (CR)^46^. These regions create critical networks where antigens, APCs/DCs, and T cells interface to initiate and direct antigen-specific immune processes, including suppression and tolerance^47,48^. At 2 days, Ea presentation localized with HEVs was detected in treated LNs (Fig. 6e), and increased by day 6 (Fig. 6f). Likewise, at day 2, Ea presentation localized with HEV was also detected in the early-draining (Fig. 6g) and late-draining (Fig. 6h) LNs; by 6 days the signal in the late-draining LNs remained elevated (Fig. 6h).

Since antigen presentation was confirmed in untreated LNs after *iLN* injection, and MPs were restricted to treated LNs, we next tested if antigen trafficking may be through lymphatic drainage following release from MPs, or through migration of APCs that internalize MPs. Mice were immunized with Ea MPs or Ea/Rapa MPs, and then APC egress from LNs was inhibited by administration of FTY720^49^—an antagonist of the S1P receptor—using three daily *i.p*. injections starting on the day of MP treatment (Fig. 6i). 1 day after the final FTY720 injection, treated and draining LNs were analyzed for Ea presentation in HEVs. In every LN analyzed, Ea and Ea/Rapa MP treatment significantly increased Ea presentation in treated (Fig. 6j), early-draining (Fig. 6k) and late-draining LNs (Fig. 6l) compared to the non-injected control. FTY720 treatment did not significantly affect Ea presentation (Fig. 6j–l) by either MP treatment. This discovery suggests trafficking of antigen from MP-treated LNs to distant LNs is not reliant on migration of APCs from the treated LNs, and instead results from lymphatic drainage.

Here we’ve demonstrated a single *iLN* injection promotes potent tolerance through concentrating delivered cargo in LNs. Such capabilities could provide significant potential for dose sparing compared to current systemic clinical regimens^50,51^. While the presentation of antigen without the presence of Rapa in untreated LNs has the potential to prime antigen-specific T cells with inflammatory function, the fact that *iLN* treatment with MPs encapsulating antigen alone does not significantly alter disease in T1D (Fig. 3b), or allogenic islet transplantation compared to vehicle controls (Fig. 3e) indicates activation of antigen-specific T cells with pro-inflammatory potential is not of significant concern. While biodistribution of Rapa after MP treatment requires further study, inhibition of mTOR signaling in untreated tissues of p31/Rapa MP treated mice (Fig. 5c, d) likely indicates dissemination of Rapa to these LNs, similar to antigen. The concentrations of antigen and Rapa accumulating in untreated LNs, and their impact on the tolerogenic immune response is unclear. However, we have previously demonstrated the co-delivery of immunostimulatory adjuvants and antigen using the *iLN* platform with MPs promoted superior antigen-specific T cell response compared to treatment with LN nanoparticles (NPs) sufficiently small to drain through efferent lymphatics^29^. Therefore, we hypothesize prolonged retention of delivered cargo in treated LNs after MP treatment is the main driver for induction of tolerance. An additional key advantage of the *iLN* platform is the ability to deliver the entire dosage of immune signals to the lymphatic system in order to promote antigen-specific T_REG_ in LNs without exposing peripheral tissue to high-dose immunosuppressants. While particulate delivery of antigen can enter the lymphatics and reach multiple LNs after peripheral administration, a large fraction of payload is retained at the injection site or can reach the non-lymphoid tissues depending on particle size and properties^38,52^. *iLN* microparticle delivery can improve these shortcomings.

In these studies we have used a non-surgical technique to inject peripheral LNs which do not drain the pancreatic islets. It has been demonstrated that LNs differ in their propensity to promote tolerogenic or inflammatory T cell responses and to imprint T cell migratory potential based on their location and drainage patterns. Priming in skin draining LNs drives T cell migration to the skin via CCL10 expression^53^, while T cell priming in gut draining LNs drives T cell migration to the gut in a retinoic acid dependent manner^54^. A previous report has shown proximal gut draining LNs preferentially promote tolerogenic responses, where distal gut draining lymph nodes promote inflammatory responses toward the same antigen^55^. Therefore, future work should investigate whether selection of LN to inject will impact efficacy in promoting tolerance. In addition, future studies should investigate the ability of MP-induced antigen-specific T_REG_ to migrate to the pancreatic islets following peripheral LN injection. In clinical settings it may be advantageous to inject islet-draining LNs when accessible during surgical islet transplantation. However, since antigen (Fig. 6e–h) and most likely Rapa, as discussed above, disseminate to other LNs along with antigen-specific T_REG_ (Fig. 5k) after *iLN* MP treatment, non-surgical injection of peripheral LNs may provide similar efficacy while obviating the need for surgery and will allow for future boosts if needed. In fact, clinical trials utilizing *iLN* injections of free islet autoantigens have injected inguinal LNs non-surgically with ultrasound guidance^27^.

### LN delivery of MPs promote a tolerogenic LN microenvironment

**Fig. 7:**
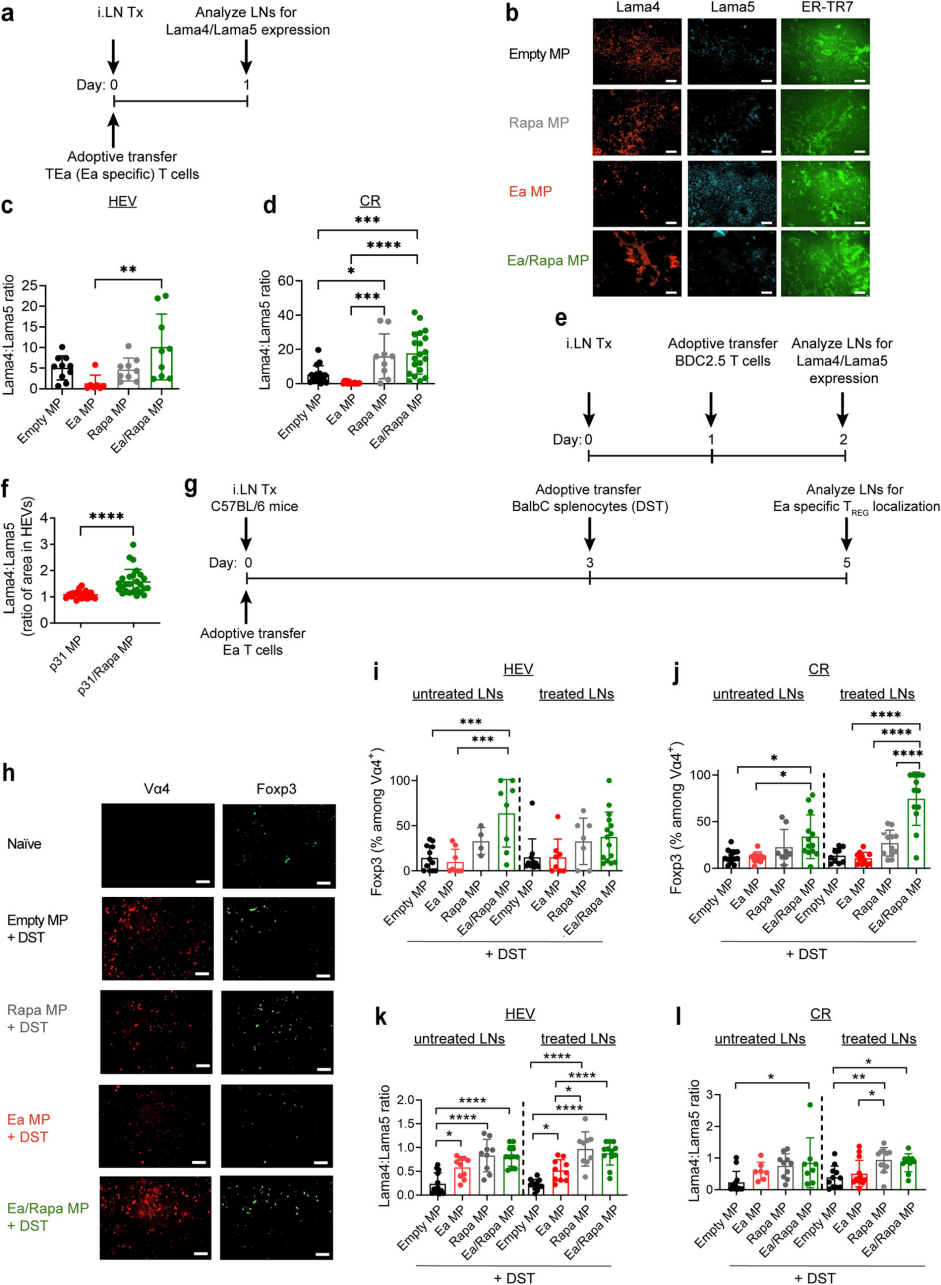
MP treatment promotes development of tolerogenic microdomains in LNs. **a** Schematic representation of experiment in panels **b**–**d**. **b** Representative immunofluorescence images of treated LNs stained for laminin α4, laminin α5, ER-TR7, and DAPI are shown; Scale bar = 50 μm. Quantification of ratio of laminin α4 to laminin α5 in **c** high endothelial venules and **d** cortical ridges in treated LNs. *N* = 4 mice for each treatment group with 2 or 3 sections per LN. One-way ANOVA with Tukey’s post hoc test was used to compare treatment groups. **e** Schematic representation of experiment in panel **f**. **f** Ratio of laminin α4 to laminin α5 in high endothelial venules of LNs. *N* = 4 mice for each treatment group with 2 or 3 sections per LN. Two-tailed Welch’s *t* test was used to compare groups, and inguinal, axillary and pancreatic LNs were pooled for each treatment group. **g** Schematic representation of experiments in panels **i**–**l**. **h** Representative immunofluorescence images showing Foxp3 expression among antigen-specific T cells in treated LNs. The naïve group did not receive adoptive transfer of Ea T cells. DAPI (Blue) and ER-TR7 (Cyan) are included in the merge: Scale bar = 50 μm. LNs were stained for Foxp3, Vα4 T cell receptor clonotype, ER-TR7, and DAPI. Representative merged images are show in Supplementary Fig. 10. Quantification of Foxp3 expression among antigen-specific T cells in **i** high endothelial venules (HEV) and **j** cortical ridges (CR). Quantification of ratio of laminin α4 to laminin α5 in **k** high endothelial venules and **l** cortical ridges in treated and untreated LNs. *N* = 4 mice for each treatment group with 2 or 3 sections per LN. Iliac LNs were used as untreated LNs. One-way ANOVA with Tukey’s post hoc test were used to compare each treatment group within individual lymph nodes in each region of the LN analyzed. For all experiments mean ± s.d. is shown. *p < 0.05, **p < 0.01, ***p < 0.001, ****p < 0.0001. Source data are provided as a Source Data file. Intra-lymph node (*iLN*).

Using genetic knock out models, we have shown during tolerance, structural changes develop in LN stroma. These are characterized by increases in the ratio of laminin α4 (lama4) to laminin α5 (lama5) locally in the HEVs and CRs of LNs^45,56,57^. In these regions, higher lama4:lama5 ratio promotes T_REG_ induction and accumulation, while lower ratios favor differentiation of T_H_1 and T_H_17 phenotypes^56^. Thus, we tested if local delivery of MPs containing self-antigen and Rapa promote tolerogenic microdomains and local T_REG_ polarization in treated LNs. Mice were immunized with Ea/Rapa MPs followed by adoptive transfer of TEa T cells. One day after T cell transfer, treated LNs were analyzed for lama4 and lama5 expression in HEVs and CR (Fig. 7a, Supplementary Fig. 10a). HEVs and CRs were identified based on morphology of the stromal fibers, ER-TR7, which are produced by fibroblastic reticular cells in CRs and encase HEVs^45^. In both HEVs (Fig. 7b, c, Supplementary Fig. 11a–c) and CR (Fig. 7b, d, Supplementary Fig. 11d, e), Ea/Rapa MPs promoted the highest lama4:lama5 ratio. In CRs Rapa MPs but not Ea MPs also significantly increased lama4:lama5 ratio, highlighting the modulatory cue as the key driver of structural rearrangement. Similar results were observed in NOD mice treated with p31/Rapa MPs and infused with BDC2.5 T cells (Fig. 7e); lama4:lama5 ratios were significantly increased in HEVs when comparing p31/Rapa with p31 treatment (Fig. 7f). These data demonstrate co-treatment with Rapa and antigen—either p31 or Ea, promotes local stromal changes in LNs associated with induction of T_REG_ response.

To determine if *iLN* MP-induced stromal rearrangement promotes local accumulation of antigen-specific T_REG_ in HEVs and CRs, C57BL/6 mice were immunized with Ea/Rapa MPs, followed by adoptive transfer of Ea TCR transgenic T cells. 3 days after T cell transfer, non-matched BALB/c donor-specific splenocytes (DST) exhibiting Ea were adoptively transferred *i.v*. to generate a systemic alloimmune response^45^. Two days following DST infusion, LNs were analyzed for T_REG_ accumulation in HEVs and CRs (Fig. 7g). To identify antigen-specific T_REG_, LNs were co-stained for Foxp3 and the Vα4 T cell receptor chain, which is expressed by the Ea T cells^45^. Ea/Rapa MPs promoted the greatest increase of antigen-specific T_REG_ in HEVs and CRs (Fig. 7h–j, Supplementary Fig. 10b). Interestingly, Rapa MP treatment did not promote TEa T_REG_ despite increasing lama4:lama5 ratio in HEVs of treated and untreated LNs and CRs of treated LNs (Fig. 7k, l). This is surprising since DST infusion provides a source of Ea antigen for TEa T cell priming in LNs. The necessity of delivering Ea in MPs to promote T_REG_ accumulation may be explained by prolonged retention of Ea in LNs after MP delivery compared to DST. Together these data indicate cognate-antigen/Rapa MPs promote tolerogenic microdomains in LNs in both homeostatic and inflammatory conditions.

### Rapa MPs and antigen/Rapa MPs inhibit disease transfer in therapeutic treatment regimens, while co-delivery of antigen with Rapa is required to provide durable immunosuppression in the NOD8.3 model of T1D

**Fig. 8:**
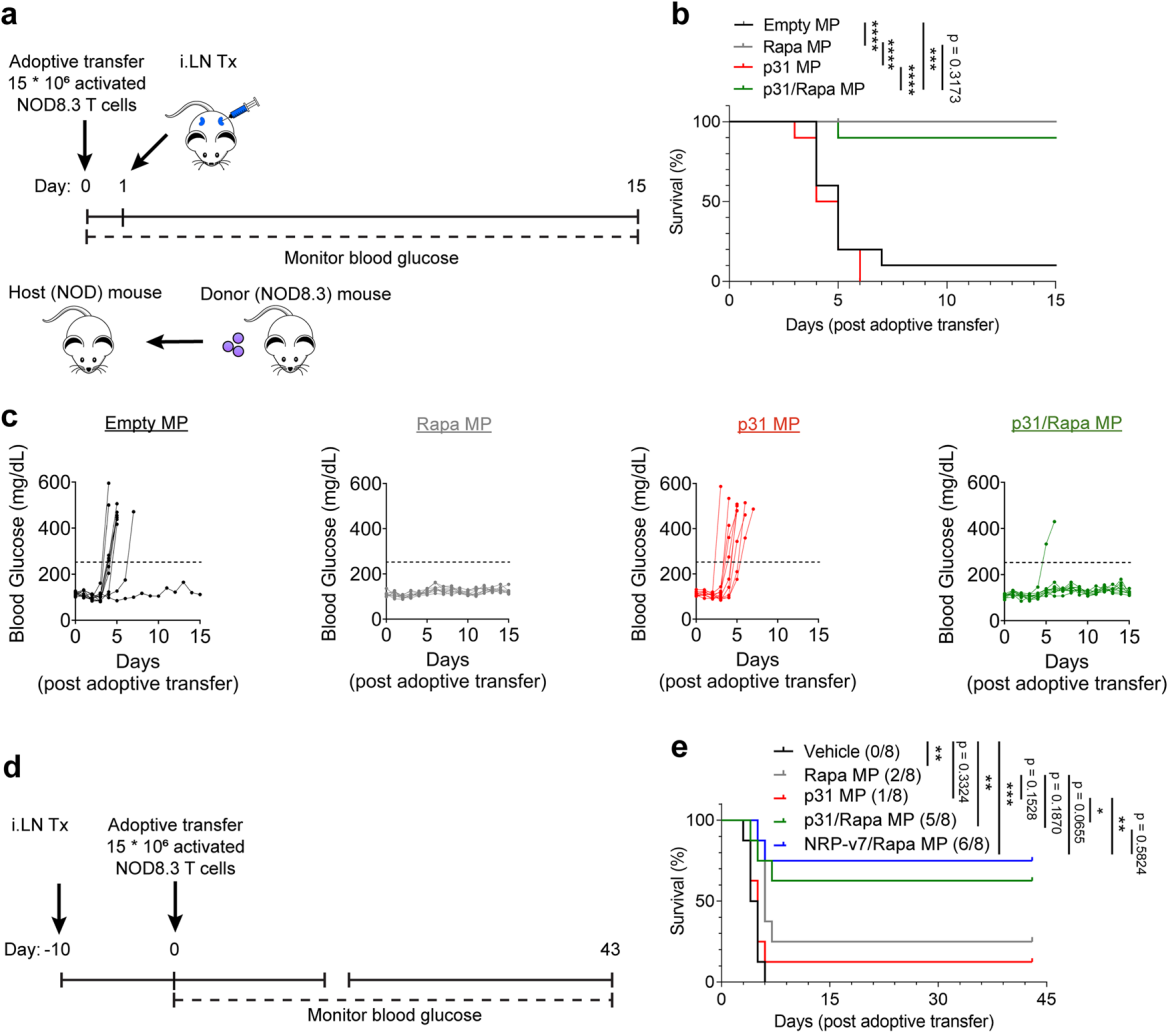
Both Rapa MP and antigen/Rapa MPs inhibit T1D induction in therapeutic regimens, while co-encapsulation of antigen with Rapa is required for durable tolerance. **a** Schematic representation of therapeutic *iLN* treatment in the NOD8.3 model of T1D. **e** Survival curve for experiment detailed in **a**. **c** Individual blood glucose traces for mice in each group. **d** Schematic representation of iLN treatment with delayed disease induction in the NOD8.3 model of T1D. **e** Survival curve for experiment detailed in **d**. The number of surviving mice in each treatment group is shown in parenthesis in the legend. *N* = 10 mice for all groups in the therapeutic treatment regimen. *N* = 8 for all treatment groups in the delayed disease induction treatment regimen. Two-tailed log-rank (Mantel-Cox) was used for all pairwise comparisons between each treatment for survival curves in **b** and **e**. *p < 0.05, **p < 0.01, ***p < 0.001, ****p < 0.0001. Source data are provided as a Source Data file. Intra-lymph node (*iLN*).

Since antigen/Rapa MPs completely inhibited disease in the NOD8.3 model when given 3 days before disease induction (Fig. 3b), we next tested if *iLN* MP treatment could promote tolerance in a more challenging therapeutic regimen. In this regimen, mice were treated *i.LN* with MPs 1 day after T cell transfer (Fig. 8a). Similar to the prophylactic treatment regimen (Fig. 3a–c), p31/Rapa MPs inhibited T1D, where 90% of mice survived for the duration of the study, while 90% of mice treated with empty MPs quickly reached humane endpoints (Fig. 8b, c). However, Rapa MPs were also highly effective in preventing disease (Fig. 8b). Together these data show in the NOD8.3 model of T1D, delivery of antigen/Rapa MPs or MPs only encapsulating Rapa is sufficient to inhibit disease when given at a timepoint close to disease induction. We hypothesized protection induced by Rapa MPs is transient, while inclusion of antigen with Rapa in MPs is necessary to promote durable tolerance.

To test whether the tolerance induced by MP treatment would be maintained even if T1D induction was performed at a much later timepoint after a single MP treatment, NOD mice were immunized 10 days before infusion of activated NOD8.3 T cells, and disease incidence was monitored daily (Fig. 8d). Excitingly p31/Rapa MPs and NRP-V7/Rapa MPs significantly improved survival relative to vehicle and p31 MP treated mice (Fig. 8e). The frequency of disease-free mice was more than doubled in both formulations combining antigen and Rapa compared to Rapa MP treatment. When benchmarking *i.LN* treatment of p31/Rapa MPs against soluble p31 and Rapa given *i.p*. at the 10 day timepoint (Supplementary Fig. 15a), *i.LN* MPs provided superior efficacy (Supplementary Fig. 15b, c). Since antigen/Rapa MPs promoted durable tolerance compared to MPs encapsulating only Rapa, we next further investigated the phenotype of MP-induced antigen-specific T_REG_.

### MPs expand antigen-specific T_REG_ with increased expression of markers of longevity

**Fig. 9:**
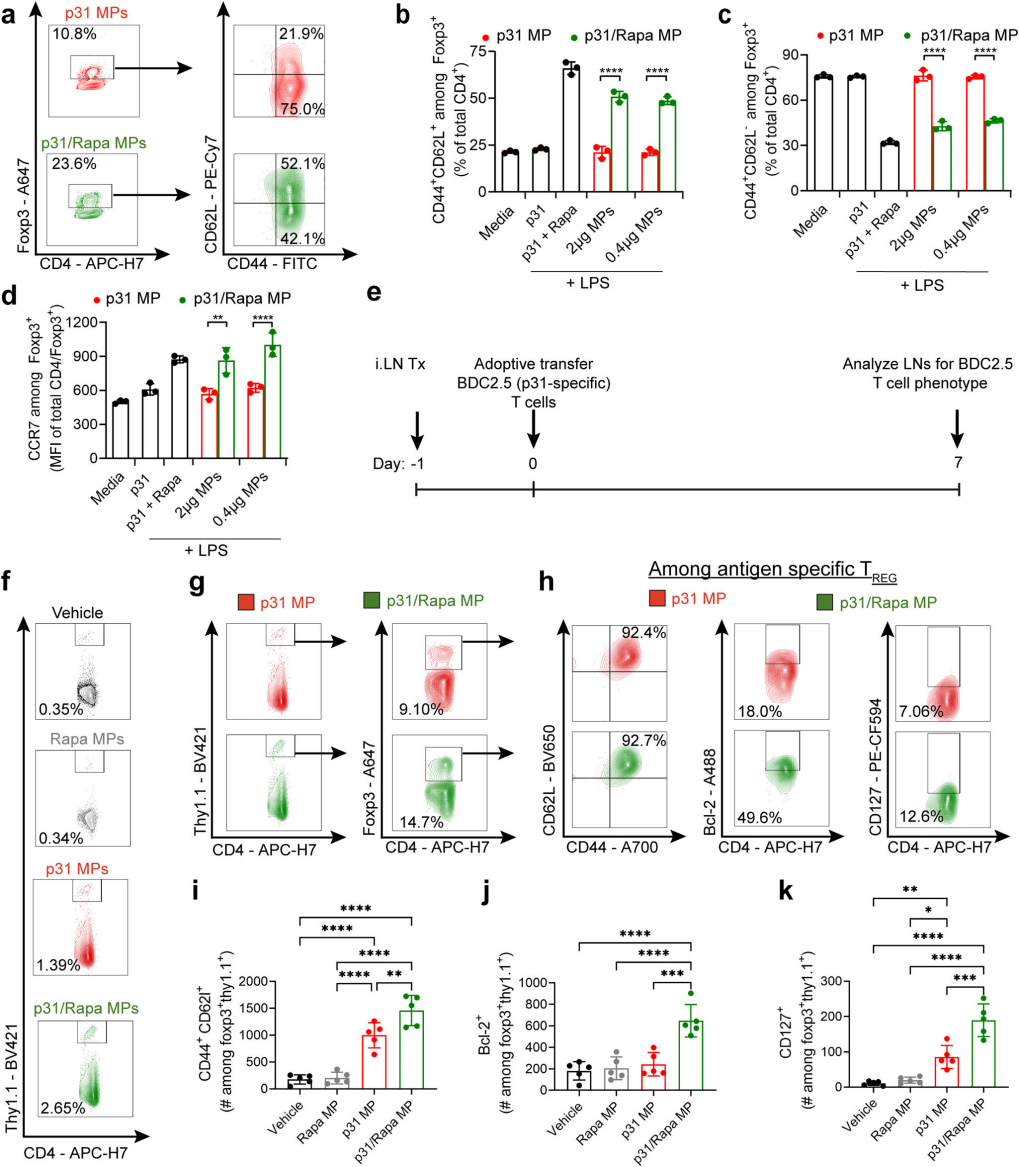
MP treatment promotes expression of memory markers among antigen-specific T_REG_ and durable tolerance in T1D. BDC2.5 T cell co-cultures were treated with p31 MPs or p31/Rapa MPs. **a** representative gating scheme to quantify co-expression of CD44 and CD62l among BDC2.5 T_REG_. Quantification of **b** CD44^+^CD62L^−^ and **c** CD44^+^CD62L^−^ frequencies T_REG_ and (**d**) mean fluorescence intensity of CCR7 among BDC2.5 T_REG_. *N* = 3 for each sample. One-way ANOVA with Tukey’s post hoc test was used to compare all groups. Comparisons for p31 MP and p31/Rapa MPs at matched doses are indicated in plots. **e** Schematic representation of BDC2.5 T cell adoptive transfer experiment in panels **f**–**k**. *N* = 5 for each treatment group. **f** Representative flow cytometry plots showing BDC2.5 T cell frequency in treated LNs. Gating scheme for **g** p31 specific T_REG_ and **h** co-expression of CD44 and CD62L, and expression of BCL-2 and CD127 among p31 specific T_REG_. Quantification of **i** co-expression of CD44 and CD62L, and expression of **j** BCL-2 and **k** CD127 among p31 specific T_REG_ in treated LNs. Treated LNs were pooled for each mouse. One-way ANOVA with Tukey’s post hoc test was used to compare all groups. For all experiments mean ± s.d. is shown. *p < 0.05, **p < 0.01, ***p < 0.001, ****p < 0.0001. Source data are provided as a Source Data file. Intra-lymph node (*iLN*).

Recent studies have demonstrated mTOR pathway inhibition during expansion of naïve T cells polarizes central memory phenotype among T_REG_, which display increased persistence in vivo^58^. Since antigen presentation after *iLN* treatment was prolonged (Fig. 6f–h), we posited T_REG_ responses polarized during mTOR inhibition may also be durable and create tolerogenic memory that has therapeutic significance in the context of autoimmune therapy. To test whether inclusion of Rapa in MPs enhances memory-like phenotype among self-reactive T_REG_, we first co-cultured DCs from NOD mice with BDC2.5 T cells in the presence of p31 MPs or p31/Rapa MPs. After 3 days T cells were stained for Foxp3 and markers characteristic of central memory T cells: CD44^+^, CD62L^+^, and CCR7 (Supplementary Fig. 12). CD44 is a marker expressed by activated T cells; CD62L and CCR7 drive homing to LNs, where long-lived memory T cells persist^59,60^. Excitingly, co-cultures treated with p31/Rapa MPs increased the frequencies of T_REG_ co-expressing CD62L and CD44 (Fig. 9a–c), as well as CCR7 (Fig. 9d). These in vitro results demonstrate inclusion of Rapa in MPs promotes antigen-specific T_REG_ with central memory phenotypes.

We next analyzed the effects of p31/Rapa MP treatment on the expression of central memory markers among antigen-specific T_REG_ in vivo. NOD mice were immunized with p31/Rapa MPs, followed by adoptive transfer of BDC2.5 T cells (Fig. 9e). 7 days after transfer, LNs were analyzed for expression of CD44, CD62L, as well as CD127 and Bcl-2 among antigen-specific Foxp3^+^ T_REG_ (Fig. 9e, Supplementary Fig. 13) in treated LNs. CD127 is the receptor for IL-7, an important survival signal for memory T cells within LNs, and Bcl-2 preserves survival by inhibiting apoptosis^61,62^. LNs treated with p31/Rapa MPs retained significantly higher frequencies of antigen-specific T cells 1 week after adoptive transfer (Fig. 9f, Supplementary Fig. 14a, b), and among these cells a higher number (Supplementary Fig. 14c) and frequency of T_REG_ (Fig. 9g, Supplementary Fig. 14d). Excitingly, the number of antigen-specific T_REG_ expressing CD44^+^CD62L^+^ (Fig. 9i), Bcl-2 (Fig. 9j) and CD127 (Fig. 9k) was significantly higher in p31/Rapa MP treated LNs compared to all other treatment groups. In addition to absolute numbers, the frequency of Bcl-2 and CD127 expressing antigen-specific T_REG_ was higher when comparing p31/Rapa MP treated LNs with p31 MP treated LNs (both treatment groups which expanded antigen-specific T cells) (Supplementary Fig. 14e–g). Similar results were observed among the total antigen-specific CD4 T cell population (Supplementary Fig. 14h–j). Thus, p31/Rapa MP treatment expands antigen-specific T_REG_ in LNs and increases expression of markers associated with durability and regulatory memory. Taken together, Fig. 9 indicates LN treatment of MPs co-encapsulating islet antigen and Rapa expands antigen-specific T_REG_ with a memory-like phenotype and supports co-delivery of antigen with Rapa is required for durable tolerance in T1D.

Several possibilities may explain increased expansion of p31-specific T cells after treatment with p31/Rapa MP compared to p31 MP (Figs. 5g and 9f). Antigen-specific T cell proliferation may be more sustained after p31/Rapa MP treatment. In addition, reduced mTOR signaling during antigenic stimulation of T cells may drive a more durable phenotype for persistence and avoiding cell death. Previous reports have established mTOR inhibition to promote central memory phenotypes among cytotoxic T cells ^63–65^; however, only recently have the effects of mTOR inhibition on T_REG_ memory phenotype^66^ been explored. Recent work has demonstrated T_REG_ in lymphoid tissue with low mTOR activity display increased markers of memory phenotype (Bcl2^high^, CD62L^high^, KLRG1^low^, PD-1^low^), lower levels of aerobic glycolysis, higher levels of oxidative phosphorylation and prolonged survival—all characteristics of long lived memory T cells^58^. While multiple reports have investigated co-delivering mTOR inhibitors with antigen to promote antigen-specific T_REG_^17,30,67^, this is the first report investigating the memory phenotype generated using this strategy. Here, p31/Rapa MP induced antigen-specific T_REG_ expressed higher levels of markers associated with longevity (Fig. 9h, j, k), and p31/Rapa MP treatment provided superior protection from T1D when disease was induced at a late timepoint following treatment (Fig. 8e). Together these data support *iLN* treatment of tolerogenic MPs promote a durable and functional antigen-specific T_REG_ response. Future studies will investigate the effects of p31/Rapa MPs on antigen-specific T_REG_ survival and suppressive function over longer time periods.

Here we demonstrate direct LN injection of MPs co-encapsulating Rapa with antigens targeted in autoimmune disease and alloimmune response is a potent therapeutic strategy to promote antigen-specific tolerance. MP treatment dramatically increased survival in models of T1D and islet transplantation mediated by both CD4 and/or CD8 pathology. Co-encapsulation of both MP components is necessary for optimal therapeutic efficacy. Mechanistically, inclusion of antigen is required to prime antigen-specific T cells in LNs, while Rapa functions to inhibit mTOR signaling during T cell priming. Excitingly, MPs expand T1D-relevant antigen-specific T_REG_ in treated as well as distant LNs, creating tolerogenic microdomains in LNs that promote these regulatory immune responses. Thus, while drug delivery is localized, the suppressive and tolerogenic effects become systemic. From clinical safety and efficacy standpoints, the LN and its immunomodulatory depot could be removed in the case of localized toxicity, and the recipient avoids the systemic polypharmacy of current immunosuppression which almost universally relies on potent induction immunosuppression along with a cocktail of other agents, such as glucocorticoids, calcineurin inhibitors, and nuclei acid pathways inhibitors. Finally, MP treatment induced expression of markers associated with prolonged persistence and memory function among antigen-specific T_REG_ and promoted durable tolerance in T1D. Together these data suggest a modular platform to promote lasting and selective tolerance in the context of T1D, potentially with reduced or eliminated side effects since not systemic or peripheral delivery is needed.

## Methods

### Ethics

All animal care and research was carried out using protocols approved and overseen by the University of Maryland and the University of Maryland IACUC committee (protocol R-Jan-22-04) in compliance with local, state, and federal guidelines.

### Animals

All animal care and research was carried out using protocols approved and overseen by the University of Maryland IACUC committee in compliance with local, state, and federal guidelines. NOD (NOD/shiltJ), BDC2.5 (NOD.Cg-Tg(TcraBDC2.5,TcrbBDC2.5)1Doi/DoiJ), NOD8.3 (NOD.Cg-Tg(TcraTcrbNY8.3)1Pesa/DvsJ), TEa (B6.Cg-Tg(Tcra,Tcrb)3Ayr/J) C57BL/6J and BALB/c mice were purchased from The Jackson Laboratory. BDC2.5-thy1.1 mice were obtained from the Genetically Modified NOD Mouse Core Facility, Harvard Medical School, Pathology, NRB 1052 G, 77 Avenue Louis Pasteur, Boston, MA 02115. All NOD, C57BL/6, and BALBC mice used in studies were 8–10 weeks old. All NOD-thy1.1, NOD8.3 and TEa mice used in these studies were <3 months old. All NOD mice were non-diabetic at the start of each study. Only female mice were used in all studies.

#### MP synthesis and characterization

Degradable PLGA (Sigma Aldrich) MPs were synthesized as previously described^29,30,65,68^ via double-emulsion. Briefly, an initial emulsion was generated by sonicating 80 mg 50:50 DL PLGA with 4 mg Rapa (LC labs) dissolved in 5 mL dichloromethane (Sigma-Aldrich) with 500 μL of water containing 1 mg of antigen. This initial emulsion was then homogenized with 40 mL water containing 1% w/v polyvinyl alcohol (Sigma) at 16,000 RPM for 3 min, and was allowed to evaporate overnight with stirring to allow evaporation of dichloromethane. MPs were then filtered through a 40 μm strainer (Corning Falcon) and collected via centrifugation at 5000 × g for 5 min at 4 °C. Supernatants were removed, and MPs were washed three times with 1 mL water. MPs were then resuspended in 1 mL water for in vitro and in vivo studies. Particle size was determined using an LA-950 laser diffraction analyzer (Horiba). To determine Rapa loading, a known mass of MPs was dissolved in dimethyl-sulfoxide (Sigma-Aldrich), and the absorbance at 278 nm was determined using UV/VIS spectrophotometry. Absorbance values were fit to a standard curve of known Rapa concentrations to calculate Rapa loading per mass of MPs. To determine peptide loading, Micro BCA Protein Assay Kit (Thermofisher Pierce) was used as previously described^30,69^. For preparation of fluorescently labeled MPs, 5 μL of DiR (Invitrogen) was dissolved with PLGA prior to the primary emulsion during MP synthesis.

#### In vitro release kinetics

To determine the release of microparticle cargo over time, a known mass of antigen-rapamycin microparticles was loaded into 10,000 kD MWCO dialysis tubing and placed in a 37 C 1× PBS sink condition in triplicate. At designated timepoints, an aliquot of MPs was removed from the dialysis tubing, lyophilized, then dissolved to a fixed MP concentration in 90% Acetonitrile in water. Remaining cargo in MPs was determined using high-performance liquid chromatography on a C18 column (XBridge^TM^ BEH 300, C18, 3.5 μm, 2.1 × 50 mm). p31 antigen was detected at 220 nm. Proportion released was determined with respect to loading at time 0. For rapamycin release, p31-Rapa MPs were aliquoted in sink condition of PBS and incubated at 37 C. At each timepoint, aliquots were spun at 5000 × *g* for 5 min, aspirated and dissolved in 90% Acetonitrile in water. Concentration was determined via UV-Vis at 280 nm.

#### Flow cytometry

The following antibodies were used for flow cytometry: CD4 (clone GK1.5, diluted 1:300), Thy1.1 (clone OX7, diluted 1:200), CD8 (clone 53-6.7, diluted 1:300), CD62L (clone MEL-14, diluted 1:200), CD44 (clone IM-7, diluted 1:200), CD127 (clone SB/199, diluted 1:200), Bcl-2 (clone 3F11, diluted 1:100), Foxp3 (clone MF23, diluted 1:50), CD69 (clone H1.2F3, diluted 1:300), anti-s6 pS235/S236 (clone N7-548, diluted 1:10), IFNγ (clone XMG1.2, diluted 1:50), IL-17 (clone TC11-18H10, diluted 1:50). All antibodies for flow cytometry were purchased from BD biosciences. Mice were euthanized and lymph nodes collected and processed into single-cell suspension by mechanical dissociation through a 40μm cell strainer. Cells were washed once with PBS containing 1% w/v BSA (FACS buffer) and blocked with anti CD32/CD16 (BD, Clone 2.4G2, diluted 1:25) for 10 min at room temperature. Cells were then stained with surface marker antibodies for 20 minutes at room temperature. Cells were then washed two times with FACS buffer and were either analyzed immediately or stained for intracellular markers. For intracellular stains cells were fixed and permeabilized by incubating with fix/perm buffer from the Foxp3/Transcription factor staining buffer set (eBioscience) for 40 min at 4 degrees C. Cells were stained with antibodies against intracellular markers for 30 min at 4 degrees C, followed by two washes with perm/wash buffer. Cells were analyzed with a Canto II (BD) or FACSCelesta (BD). DAPI or LIVE/DEAD Fixable Viability Dye (Thermofisher) were used for cell viability. Flow cytometry data analysis was performed using FlowJo software (version 9, Treestar).

#### In Vitro T cell co-cultures

CD11c dendritic cells were isolated from mouse splenocytes using positive magnetic selection (Miltenyi) and were plated at 100,000 cells/well in 96 well plates. For co-cultures utilizing BDC2.5 or NOD8.3 T cells, dendritic cells (DCs) were isolated from NOD mice. For co-cultures utilizing Ea T cells, DCs were isolated from C57-BL6J mice. Following isolation, DCs were stimulated with 1 μg/mL lipopolysaccharide (LPS) to aid in T cell activation and treated with indicated doses of MPs for 24 h. T cells were then isolated from splenocytes using negative magnetic selection (Stemcell), labeled with 5 μL CSFE or CellTrace Violet (Thermofisher), and 300,000 T cells were co-cultured with MP-treated DCs for 3 days. CD4^+^ enrichment was used for BDC2.5 and Ea T cells, and CD8^+^ selection was used for NOD8.3 T cells. After 3 days, co-cultures were stimulated with cell stimulation cocktail (Thermofisher) in the presence of BFA (Biolegend) for 4 h, followed by surface and intracellular marker antibody staining and analysis flow cytometry to assess proliferation and determine T cell phenotype.

#### Peptides

p31 (YVRPLWVRME), Ea (ASFEAQGALANIAVDKA) and NRP-V7(KYNKANVFL) were synthesized by Genscript with purity >98%.

#### *Intra-lymph node* injection

*iLN* injection of mice was performed as previously described^29,30,65,68,69^. Briefly hair surrounding the injection site was removed using a mild depilatory cream, and mice were injected subcutaneously at the tail base with a tracer dye (Evan’s Blue). One day after tracer dye injection, both inguinal LNs were identified and injected with 1 mg of MPs, unless otherwise stated. MPs were suspended in 10 μL sterile water and injected *iLN* using a 31 g insulin syringe.

#### *Intra-peritoneal* injection with soluble p31 and soluble rapamycin

NOD mice were injected *i.p* at the indicated treatment times and doses with soluble p31 and soluble rapamycin in 100 μL of 5% DMSO in PBS.

#### Diabetes induction through NOD8.3 T cell adoptive transfer

8 week old female NOD mice were immunized *iLN* with indicated MP treatments. Splenocytes from NOD8.3 mice were stimulated with 1 μM NRP-V7 peptide at 2 × 10^6^ cells/mL for 3 days, washed with PBS, and injected *i.v*. into host NOD mice at 15 × 10^6^ cells/mouse. Blood glucose was monitored daily following T cell infusion, and mice were considered diabetic at a blood glucose level >250 mg/dL.

#### Islet transplantation studies

8–10 week old C57BL/6 mice were induced with diabetes by intra-peritoneal injection of 180 mg/kg Streptozotocin. Islets were implanted and lymph nodes were injected with indicated treatments on the day host mice became diabetic. The time interval (Δt) between Streptozotocin and islet transplantation was 7–14 days for all mice in the study. To collect allogenic islets, BALB/c mice were euthanized and the common bile duct was injected with 3 mL cold Hanks’ buffer containing 1.5 mg/mL of collagenase-P (Roche Diagnostics, Indianapolis, IN). Pancreata was surgically excised and digested at 37 °C for 15 min. The digested pancreas was disrupted by vigorous shaking, and the suspension was washed twice with RPMI 1640 containing 10% fetal bovine serum. Pancreatic islet separation was performed by centrifugation on a discontinuous Ficoll (Sigma) gradient of 11%, 21%, 23%, and 25%. Islets were picked from the second layer, and 400 islets were transplanted beneath the renal capsule of host mice. Blood glucose was monitored daily, and mice were euthanized after two consecutive readings of blood glucose >300 mg/dL.

#### BDC2.5 T cell adoptive transfer studies

8–10 week old female NOD mice were immunized *iLN* with indicated MP treatments. One day following *iLN* treatment, BDC2.5 T cells were isolated from splenocytes of BDC2.5 mice BDC2.5-thy1.1 mice using CD4 negative magnetic selection (Stemcell) per the manufacturer’s instructions and were adoptively transferred to host NOD mice T cells/mouse. In studies using BDC2.5 T cells, cells were labeled with CSFE prior to adoptive transfer. At indicated times following T cell transfer, host mice were sacrificed and LNs were processed and analyzed by flow cytometry. BDC2.5 T cells were identified based on CSFE signal when using BDC2.5 T cells or the expression of thy1.1 when using BDC2.5-thy1.1 T cells.

### In vivo imaging

Imaging of indicated surgically excised LNs was performed using a Perkin-Elmer IVIS Spectrum in vivo imaging system. DiR (Thermo) Fluorescent MPs were synthesized using MP double emulsion described with 0.4 mg DiR added to the organic polymer solution. Animals were injected *iLN* with MPs as described with either empty (non-fluorescent), or Vehicle MPs. 2 and 4 days following *iLN* injection, mice were euthanized and LNs were excised and imaged. Exposure times and lamp settings were determined from single-fluorophore controls. Image analysis was performed using Living Image Software (Perkin Elmer) and quantitative analysis was done using doing region of interest (ROI) analysis of total radiant efficiency. Number of mice and LNs imaged are indicated in figure legends.

### Immunohistochemistry and histology

The following primary antibodies were used for immunohistochemistry diluted 1:200: ERTR7 (scbt, clone sc-73355), CD11c (BD, clone HL3), Laminin alpha 4 (Novus Bio, clone 775830), laminin alpha 5 (Novus Bio, polyclonal), Foxp3 (ThermoFisher, clone NRRF-30), Y-Ae (scbt, clone sc-32247). For immunohistochemistry, LNs and spleens were frozen with OCT compound (Scigen Tissue-Plus). Frozen sections were cut at 6μm, fixed with cold acetone, blocked with 5% goat or donkey serum (Jackson ImmunoResearch, West Grove, PA) and incubated with the indicated antibodies and DAPI. Samples were incubated with secondary antibodies diluted 1:400 for 1 h (Rabbit IgG, Goat polyclonal, Jackson Immunoresearch; Rat IgG Goat Polyclonal, Jackson Immunoresearch; Rabbit IgG, Donkey Polyclonal, Jackson Immunoresearch; Rat IgM, Goal Polyclonal Jackson Immunoresearch). Samples were further processed as described previously^45,46,56^. Images were acquired with a Nikon Eclipse 700 (Nikon, Melville, NY, USA) and analyzed with Volocity image analysis software (version 4.7.2) Perkin Elmer, Waltham, MA). The positive staining area percentage was quantified based on at least three independent experiments with 3 sections per LN and 3–5 fields/section. Number of mice are indicated in figure legends.

#### Statistics and reproducibility

Analyses were carried out with Graphpad Prism (version 9.3.1). The specifics of statistical tests for each experiment and the number of replicates are detailed in figure legends. Error bars in all panels represent the mean ± standard deviation and *p* values ≤ 0.05 were considered significant with levels of significance were defined as *p < 0.05, **p < 0.01, ***p < 0.001, ****p < 0.0001. For comparisons of more than two groups, one-way ANOVA with Tukey’s post test was performed. For comparisons of two groups, two-tailed Welch’s *t* test was performed. For T1D and allogenic islet transplantation survival studies, two-tailed log-rank (Mantel-Cox) was used for all pairwise comparisons between each treatment for survival curves. For experiments using immunohistochemistry and histology, representative images of data from two similar experiments are shown.

### Reporting summary

Further information on research design is available in the Nature Portfolio Reporting Summary linked to this article.

## Supplementary information

Supplementary Information

Reporting Summary

## Data Availability

All relevant data supporting the key findings of this study are available within the article and its Supplementary Information files or from the corresponding author upon reasonable request. Source data are provided as a Source Data file. Source data are provided with this paper.

## Source data

Source Data

## References

[1] Schofield J, Ho J, Soran H (2019). Cardiovascular risk in type 1 diabetes mellitus. Diabetes Ther..

[2] Khoshbaten M, Madad L, Baladast M, Mohammadi M, Aliasgarzadeh A (2011). Gastrointestinal signs and symptoms among persons with diabetes mellitus. Gastroenterol. Hepatol. Bed Bench.

[3] Fugger L, Jensen LT, Rossjohn J (2020). Challenges, progress, and prospects of developing therapies to treat autoimmune diseases. Cell.

[4] Hansel TT, Kropshofer H, Singer T, Mitchell JA, George AJT (2010). The safety and side effects of monoclonal antibodies. Nat. Rev. Drug Discov..

[5] Masharani UB, Becker J (2010). Teplizumab therapy for type 1 diabetes. Expert Opin. Biol. Ther..

[6] Herold KC (2019). An anti-CD3 antibody, teplizumab, in relatives at risk for type 1 diabetes. N. Engl. J. Med..

[7] Sims EK (2021). Teplizumab improves and stabilizes beta cell function in antibody-positive high-risk individuals. Sci. Transl. Med..

[8] Cayabyab F, Nih LR, Yoshihara E (2021). Advances in pancreatic islet transplantation sites for the treatment of diabetes. Front. Endocrinol..

[9] Ricordi C (1992). Human islet isolation and allotransplantation in 22 consecutive cases. Transplantation.

[10] Nakamura T (2020). Long-term outcome of islet transplantation on insulin-dependent diabetes mellitus: an observational cohort study. J. Diabetes Investig..

[11] Gammon JM, Jewell CM (2019). Engineering immune tolerance with biomaterials. Adv. Healthc. Mater..

[12] Gosselin EA, Eppler HB, Bromberg JS, Jewell CM (2018). Designing natural and synthetic immune tissues. Nat. Mater..

[13] Froimchuk E, Oakes RS, Kapnick SM, Yanes AA, Jewell CM (2021). Biophysical properties of self-assembled immune signals impact signal processing and the nature of regulatory immune function. Nano Lett..

[14] Oakes RS (2021). Exploiting rational assembly to map distinct roles of regulatory cues during autoimmune therapy. ACS Nano.

[15] Tostanoski LH, Eppler HB, Xia B, Zeng X, Jewell CM (2019). Engineering release kinetics with polyelectrolyte multilayers to modulate TLR signaling and promote immune tolerance. Biomater. Sci..

[16] Tostanoski LH (2016). Design of polyelectrolyte multilayers to promote immunological tolerance. ACS Nano.

[17] Maldonado RA (2015). Polymeric synthetic nanoparticles for the induction of antigen-specific immunological tolerance. Proc. Natl Acad. Sci. USA.

[18] Saito E (2020). Modulating lung immune cells by pulmonary delivery of antigen-specific nanoparticles to treat autoimmune disease. Sci. Adv..

[19] Kenison JE (2020). Tolerogenic nanoparticles suppress central nervous system inflammation. Proc. Natl Acad. Sci. USA.

[20] Yeste A (2016). Tolerogenic nanoparticles inhibit T cell-mediated autoimmunity through SOCS2. Sci. Signal..

[21] Sharabi A (2018). Regulatory T cells in the treatment of disease. Nat. Rev. Drug Discov..

[22] Jain A, Pasare C (2017). Innate control of adaptive immunity: beyond the three-signal paradigm. J. Immunol..

[23] Senti G (2008). Intralymphatic allergen administration renders specific immunotherapy faster and safer: a randomized controlled trial. Proc. Natl Acad. Sci. USA.

[24] Werner MT, Bosso JV (2021). Intralymphatic immunotherapy for allergic rhinitis: a systematic review and meta-analysis. Allergy Asthma Proc..

[25] Johansen P (2005). Direct intralymphatic injection of peptide vaccines enhances immunogenicity. Eur. J. Immunol..

[26] Ribas A (2011). Intra–Lymph Node Prime-Boost Vaccination against Melan A and Tyrosinase for the Treatment of Metastatic Melanoma: Results of a Phase 1 Clinical Trial. Clin. Cancer Res..

[27] Ludvigsson J, Wahlberg J, Casas R (2017). Intralymphatic injection of autoantigen in type 1 diabetes. N. Engl. J. Med..

[28] Casas R (2020). Glutamic acid decarboxylase injection into lymph nodes: beta cell function and immune responses in recent onset type 1 diabetes patients. Front. Immunol..

[29] Jewell CM, López SCB, Irvine DJ (2011). In situ engineering of the lymph node microenvironment via intranodal injection of adjuvant-releasing polymer particles. Proc. Natl Acad. Sci. USA.

[30] Tostanoski LH (2016). Reprogramming the local lymph node microenvironment promotes tolerance that is systemic and antigen specific. Cell Rep..

[31] Trudeau JD (2003). Prediction of spontaneous autoimmune diabetes in NOD mice by quantification of autoreactive T cells in peripheral blood. J. Clin. Invest..

[32] Wang J (2010). In situ recognition of autoantigen as an essential gatekeeper in autoimmune CD8+ T cell inflammation. Proc. Natl Acad. Sci. USA.

[33] Verdaguer J (1997). Spontaneous autoimmune diabetes in monoclonal T cell nonobese diabetic mice. J. Exp. Med..

[34] Dai YD (2005). A peptide of glutamic acid decarboxylase 65 can recruit and expand a diabetogenic T cell clone, BDC2.5, in the pancreas. J. Immunol..

[35] Kontos S, Kourtis IC, Dane KY, Hubbell JA (2013). Engineering antigens for in situ erythrocyte binding induces T-cell deletion. Proc. Natl Acad. Sci. USA.

[36] Wilson DS (2019). Synthetically glycosylated antigens induce antigen-specific tolerance and prevent the onset of diabetes. Nat. Biomed. Eng..

[37] Tang Q (2004). In vitro–expanded antigen-specific regulatory T cells suppress autoimmune diabetes. J. Exp. Med..

[38] Johansen P (2008). Antigen kinetics determines immune reactivity. Proc. Natl Acad. Sci. USA.

[39] Shah S (2019). Optimizing PLG nanoparticle-peptide delivery platforms for transplantation tolerance using an allogeneic skin transplant model. Biomaterials.

[40] Hunter Z (2014). A Biodegradable nanoparticle platform for the induction of antigen-specific immune tolerance for treatment of autoimmune disease. ACS Nano.

[41] Getts DR (2014). Therapeutic inflammatory monocyte modulation using immune-modifying microparticles. Sci. Transl. Med..

[42] Hess KL (2017). Engineering immunological tolerance using quantum dots to tune the density of self-antigen display. Adv. Funct. Mater..

[43] Carambia A (2015). Nanoparticle-based autoantigen delivery to Treg-inducing liver sinusoidal endothelial cells enables control of autoimmunity in mice. J. Hepatol..

[44] Cibrián D, Sánchez-Madrid F (2017). CD69: from activation marker to metabolic gatekeeper. Eur. J. Immunol..

[45] Warren KJ, Iwami D, Harris DG, Bromberg JS, Burrell BE (2014). Laminins affect T cell trafficking and allograft fate. J. Clin. Invest..

[46] Li L (2020). The lymph node stromal laminin α5 shapes alloimmunity. J. Clin. Invest..

[47] Burrell BE (2015). Lymph node stromal fiber ER-TR7 modulates CD4+ T cell lymph node trafficking and transplant tolerance1. Transplantation.

[48] Ochando JC (2006). Alloantigen-presenting plasmacytoid dendritic cells mediate tolerance to vascularized grafts. Nat. Immunol..

[49] Honig SM (2003). FTY720 stimulates multidrug transporter- and cysteinyl leukotriene-dependent T cell chemotaxis to lymph nodes. J. Clin. Invest..

[50] Hariharan S, Israni AK, Danovitch G (2021). Long-term survival after kidney transplantation. N. Engl. J. Med..

[51] Halloran PF (2004). Immunosuppressive drugs for kidney transplantation. N. Engl. J. Med..

[52] Almeida JPM, Chen AL, Foster A, Drezek R (2011). In vivo biodistribution of nanoparticles. Nanomed.

[53] Sigmundsdottir H (2007). DCs metabolize sunlight-induced vitamin D3 to ‘program’ T cell attraction to the epidermal chemokine CCL27. Nat. Immunol..

[54] Iwata M (2004). Retinoic acid imprints gut-homing specificity on T cells. Immunity.

[55] Esterházy D (2019). Compartmentalized gut lymph node drainage dictates adaptive immune responses. Nature.

[56] Simon T (2019). Differential regulation of T cell immunity and tolerance by stromal laminin expressed in the lymph node. Transplantation.

[57] Simon T, Bromberg JS (2017). Regulation of the immune system by laminins. Trends Immunol..

[58] Sun I-H (2018). mTOR Complex 1 signaling regulates the generation and function of central and effector Foxp3+ regulatory T cells. J. Immunol..

[59] Catron DM, Rusch LK, Hataye J, Itano AA, Jenkins MK (2006). CD4+ T cells that enter the draining lymph nodes after antigen injection participate in the primary response and become central–memory cells. J. Exp. Med..

[60] Klebanoff CA (2005). Central memory self/tumor-reactive CD8+ T cells confer superior antitumor immunity compared with effector memory T cells. Proc. Natl Acad. Sci. USA.

[61] Wojciechowski S (2007). Bim/Bcl-2 balance is critical for maintaining naive and memory T cell homeostasis. J. Exp. Med..

[62] Schluns KS, Kieper WC, Jameson SC, Lefrançois L (2000). Interleukin-7 mediates the homeostasis of naïve and memory CD8 T cells in vivo. Nat. Immunol..

[63] Araki K (2009). mTOR regulates memory CD8 T-cell differentiation. Nature.

[64] Gattinoni L, Klebanoff CA, Restifo NP (2009). Pharmacologic induction of CD8+ T cell memory: better living through chemistry. Sci. Transl. Med..

[65] Gammon JM (2017). Low-dose controlled release of mTOR inhibitors maintains T cell plasticity and promotes central memory T cells. J. Controlled Release.

[66] Smigiel KS (2013). CCR7 provides localized access to IL-2 and defines homeostatically distinct regulatory T cell subsets. J. Exp. Med..

[67] LaMothe RA (2018). Tolerogenic nanoparticles induce antigen-specific regulatory T cells and provide therapeutic efficacy and transferrable tolerance against experimental autoimmune encephalomyelitis. Front. Immunol..

[68] Andorko JI, Gammon JM, Tostanoski LH, Zeng Q, Jewell CM (2016). Targeted programming of the lymph node environment causes evolution of local and systemic immunity. Cell. Mol. Bioeng..

[69] Andorko, J. I., Tostanoski, L. H., Solano, E., Mukhamedova, M. & Jewell, C. M. Intra-lymph node injection of biodegradable polymer particles. *J. Vis. Exp.*10.3791/50984 (2014). 10.3791/50984 PMC4047663 24430972

