# Supplementary material for: Engineering the lymph node environment promotes antigen-specific efficacy in type 1 diabetes and islet transplantation
Source: Nat Commun. 2023 Feb 8;14:681. doi: 10.1038/s41467-023-36225-5 (PMC9908900; doi:10.1038/s41467-023-36225-5)
Supplement: Supplementary file 1 — Supplementary Information [file 41467_2023_36225_MOESM1_ESM.pdf]

Engineering the lymph node environment promotes antigen-specific efficacy in type 1 diabetes and islet transplantation

Supplementary Information

‡Joshua M. Gammon[1], ‡Sean T. Carey[1], Vikas Saxena[2], Haleigh B. Eppler[1], Shannon J. Tsai[1], Christina Paluskievicz[2], Yanbao Xiong[2], Lushen Li[2], Marian Ackun-Farmmer[1], Lisa H. Tostanoski[1], Emily A. Gosselin[1], Alexis A. Yanes[1], Xiangbin Zeng[1], Robert S. Oakes[1,3], *Jonathan S. Bromberg[2,4], and *Christopher M. Jewell[1,2-6]

[1] Fischell Department of Bioengineering, University of Maryland, College Park, 8278 Paint Branch Drive, College Park, MD, 20742, United States
[2] Department of Surgery, University of Maryland Medical School, 22 S. Greene Street, S8B06, Baltimore, MD, 21201, United States
[3] Department of Veterans Affairs, VA Maryland Health Care System, 10. N Green Street, Baltimore, MD 21201, USA
[4] Department of Microbiology and Immunology, University of Maryland Medical School, 685 West 30 Baltimore Street, HSF-I Suite 380, Baltimore, MD, 21201, United States
[5] Robert E. Fischell Institute for Biomedical Devices, 8278 Paint Branch Drive, College Park, MD 20742, United States
[6] Marlene and Stewart Greenebaum Cancer Center, 22 S. Greene Street, Suite N9E17, Baltimore, 32 MD 21201, United States

‡ These authors contributed equally.
Correspondence should be addressed to C.M.J. (email: cmjewell@umd.edu) and J.S.B. (email: JBromberg@som.umaryland.edu

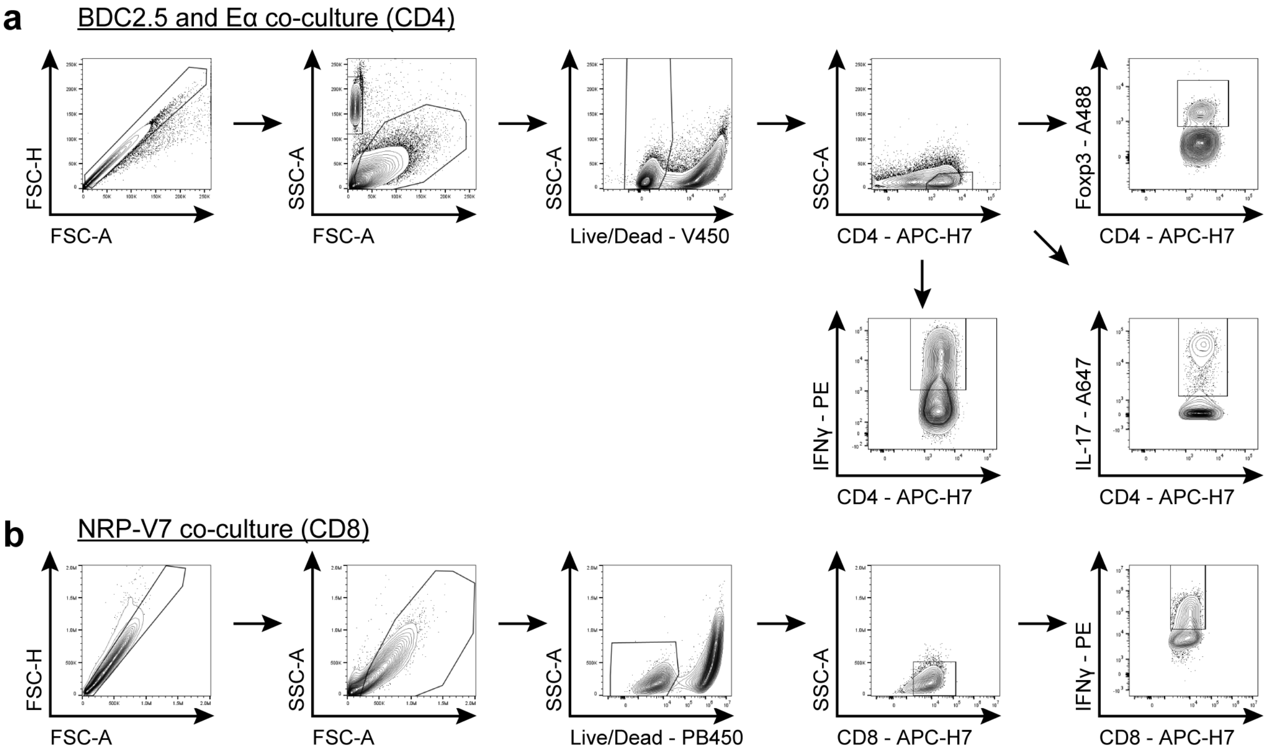

Supplementary Figure 1. Gating scheme for T cell co-culture studies with (a) p31 and Eα specific T cells and (b) NRP-V7 specific T cells detailed in **Figure 2**.

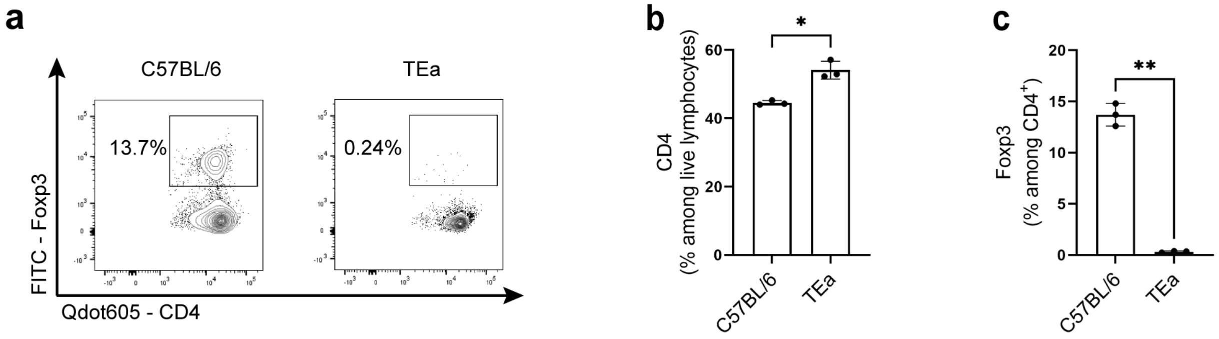

Supplemental Figure 2. TEa mice have almost no endogenous T_{REG}. Inguinal, axillary and brachial lymph nodes from C57/BL6 and TEa mice were analyzed for T_{REG} frequency by flow cytometry. (a) representative flow cytometry plots of CD4 and foxp3 expression. (b) Summary data showing T_{REG} frequency in each lymph node. N = 1, and Two tailed Welch's t test was used for statistical comparison of T_{REG} frequency. Mean ± s.d. is shown * indicates $p < 0.05$, ** indicates $p < 0.01$, *** indicates $p < 0.001$, **** indicates $p < 0.0001$

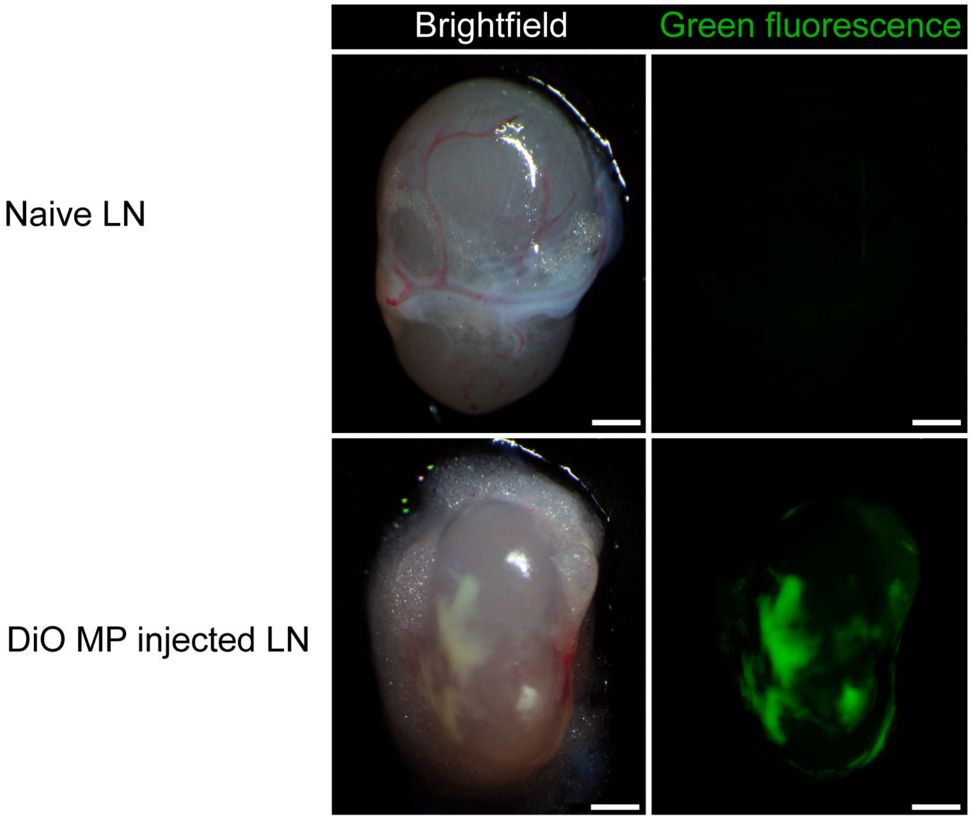

Supplemental figure 3. 1mg of MPs in 10μL water can be injected into a murine inguinal LN. An inguinal LN from a NOD mouse was excised and injected with 1mg of DIO labeled MPs in 10μL. Bright field and green fluorescent images of a naïve LN or a DIO MP injected LN are shown. * indicates $p < 0.05$, ** indicates $p < 0.01$, *** indicates $p < 0.001$, **** indicates $p < 0.0001$

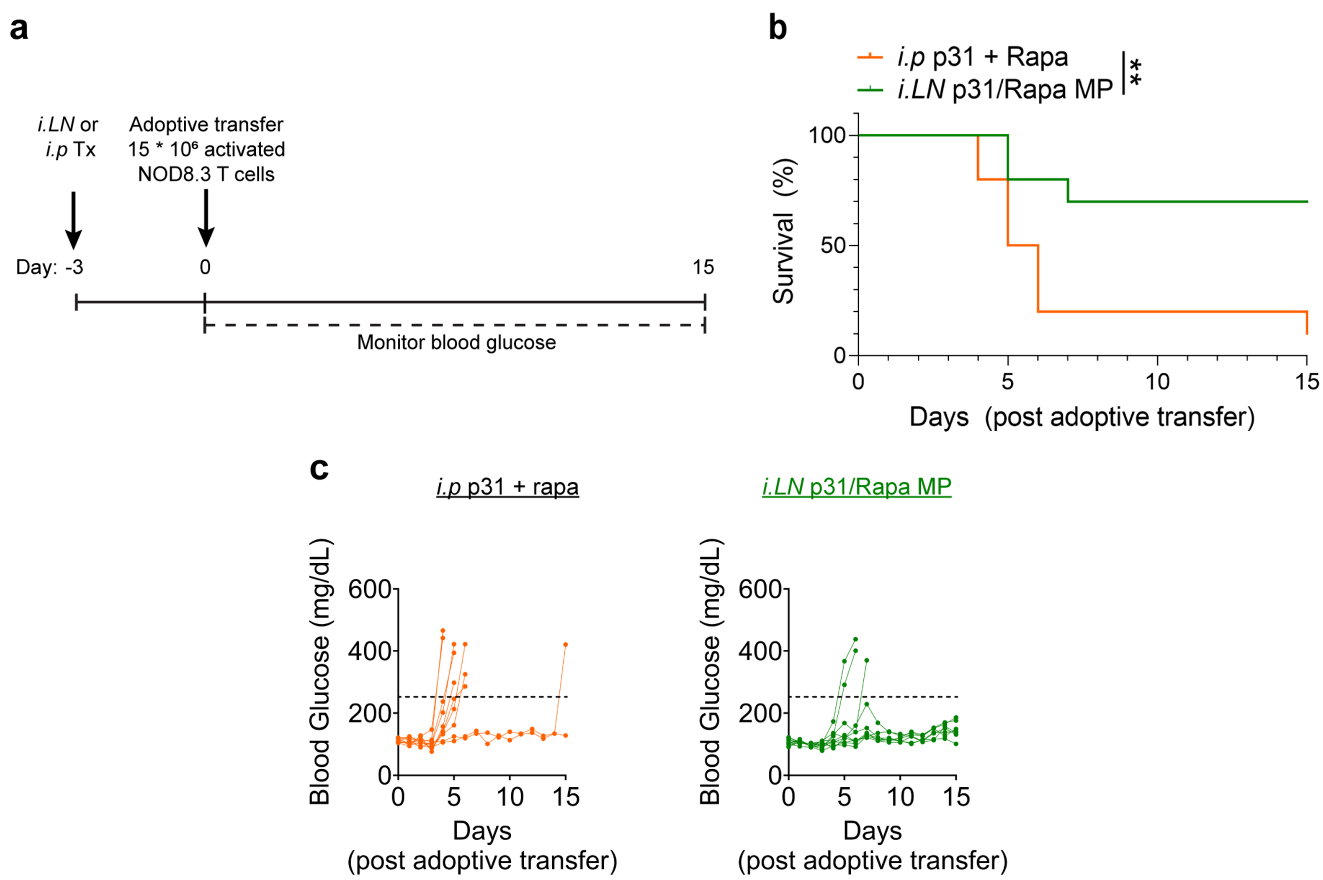

Supplementary figure 4. i.LN p31/Rapa MPs promote superior tolerance in T1D compared to soluble p31 and Rapa given *i.p.* (a) Schematic representation of NRP-V7 adoptive transfer induced T1D experiment, where host mice were treated with *i.LN* p31/Rapa MPs, or *i.p* soluble p31 with soluble Rapa. The dose for p31/Rapa MPs was 0.2mg of MPs, which is $1/10^{th}$ of the dose used in all other studies. The dose for p31 was 8µg of Rapa and 2µg of p31 for the *i.p.* treatment. N = 10 for each treatment group. (b) Survival curve indicating percent of mice remaining normoglycemic. (c) Individual blood glucose traces for mice in each group. Two tailed log-rank (Mantel-Cox) was used for all pairwise comparisons between each treatment. * indicates p < 0.05, ** indicates p < 0.01, *** indicates p < 0.001, **** indicates p < 0.0001

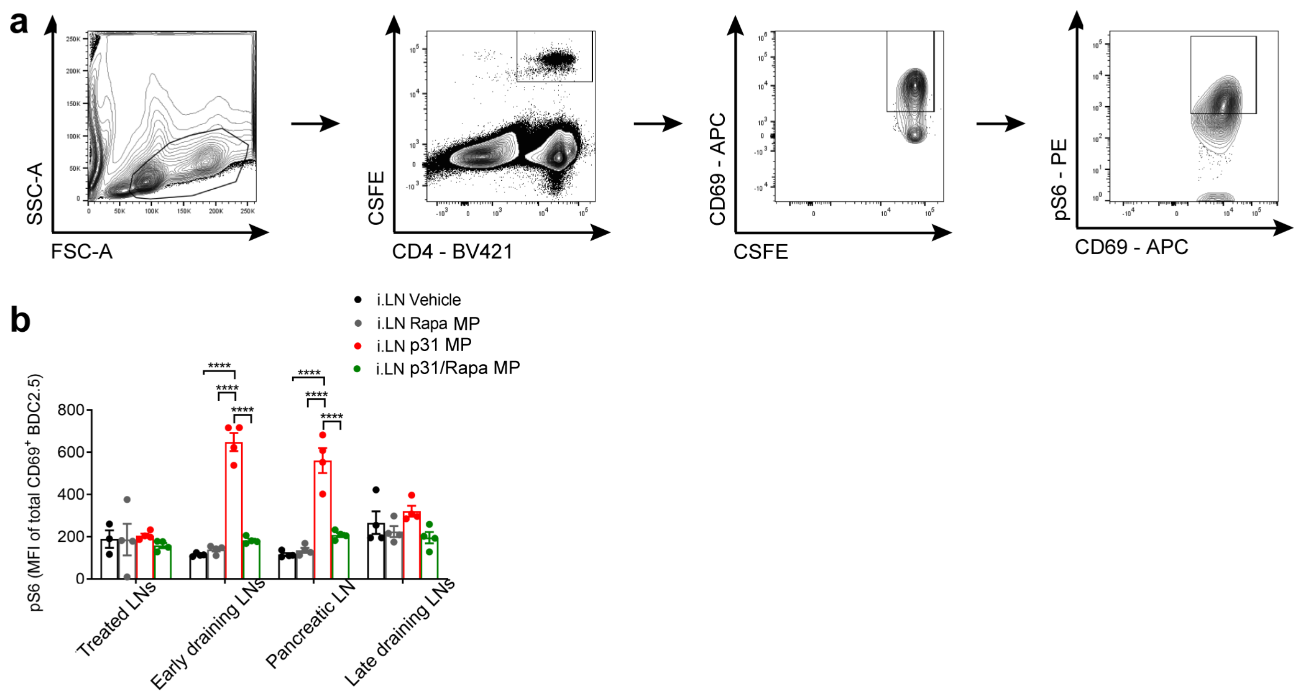

Supplementary Figure 5. (a) Gating scheme to analyze CD69 and ps6 expression among p31 specific T cells in the experiment detailed in **Figure 4**. (b) Geometric mean fluorescence intensity of ps6 among p31 specific CD69$^+$ cells from experiment detailed in **Figure 4a**. * indicates $p < 0.05$, ** indicates $p < 0.01$, *** indicates $p < 0.001$, **** indicates $p < 0.0001$

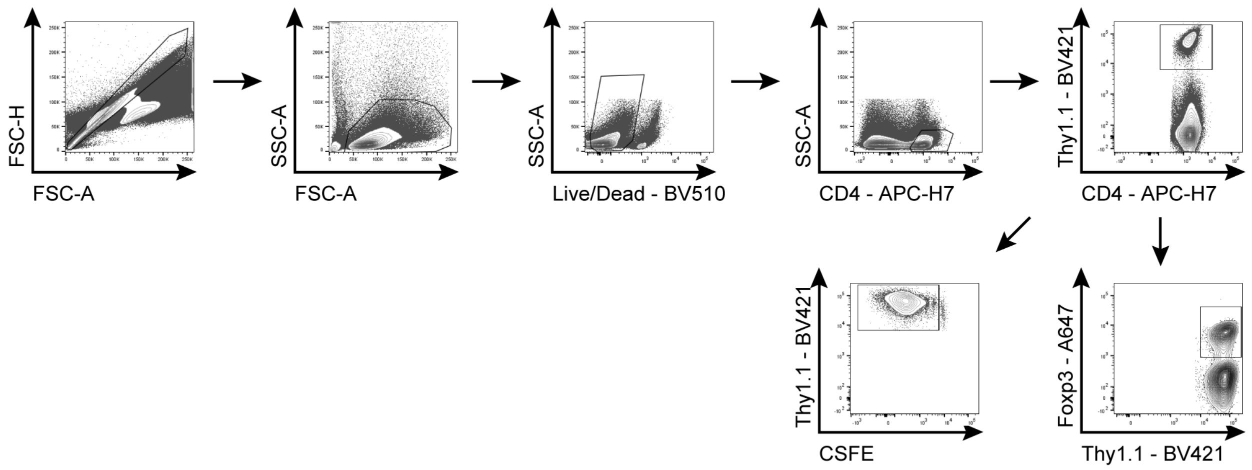

Supplementary Figure 6. Gating scheme to analyze Foxp3 expression and CSFE dilution among p31 specific T cells in the experiments detailed in **Figure 5**.

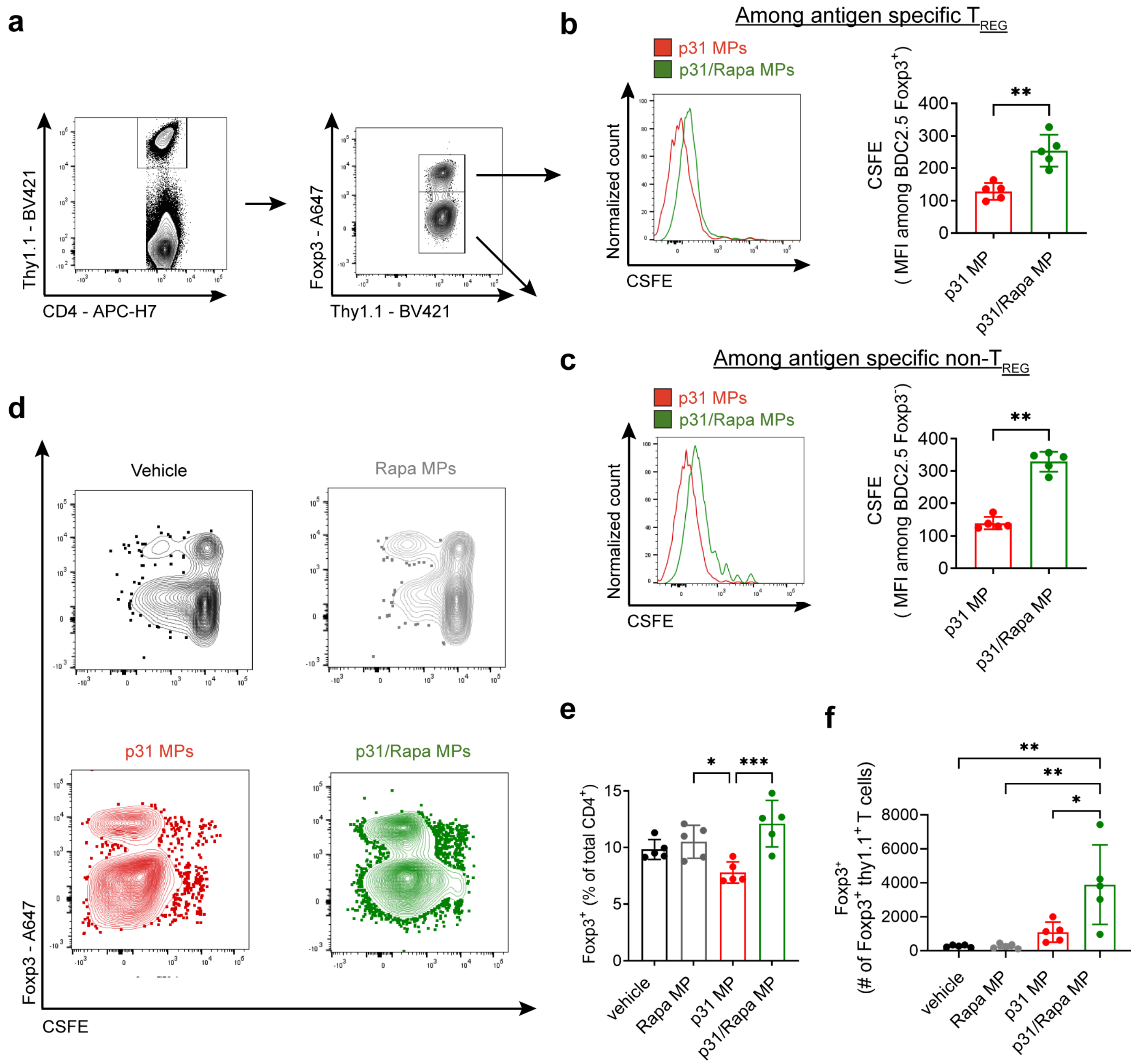

Supplementary Figure 7. Experimental details for these data are outlined in **Figure 5**. (a) Gating scheme for analysis shown in (b) and (c). (b) Representative flow cytometry traces showing CSFE dilution and average geometric mean fluorescence intensity among p31-specific T_{REG} in treated LNs of mice treated with p31 MP or p31/Rapa MPs. (c) Representative flow cytometry traces showing CSFE dilution and average geometric mean fluorescence intensity among p31-specific non-T_{REG} (determined by no Foxp3 expression) from treated LNs of mice treated with p31 MP or p31/Rapa MPs. Two tailed Welch's t test was used to compare p31 MP vs p31/Rapa MP treatment groups. (d) Representative flow cytometry plots of CSFE and Foxp3 fluorescence of antigen-specific CD4 T cells in LNs treated with vehicle, Rapa MP, p31 MP or p31/Rapa MP. (e) Frequency of T_{REG} among all CD4 T cells in treated LNs of mice treated LNs. (f) Number of antigen-specific T_{REG} in treated LNs. One way ANOVA with Tukey's post hoc test was used to compare treatment groups. * indicates $p < 0.05$, ** indicates $p < 0.01$, *** indicates $p < 0.001$, **** indicates $p < 0.0001$

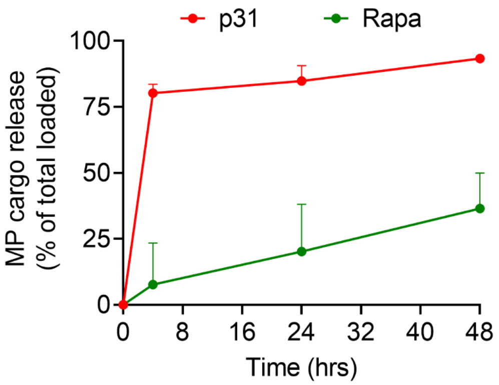

Supplementary Figure 8. Release kinetics of p31/Rapa Mps. N = 3 batches of p31/Rapa MPs. The mean ± s.d of percentage of total cargo release at each timepoint is shown.

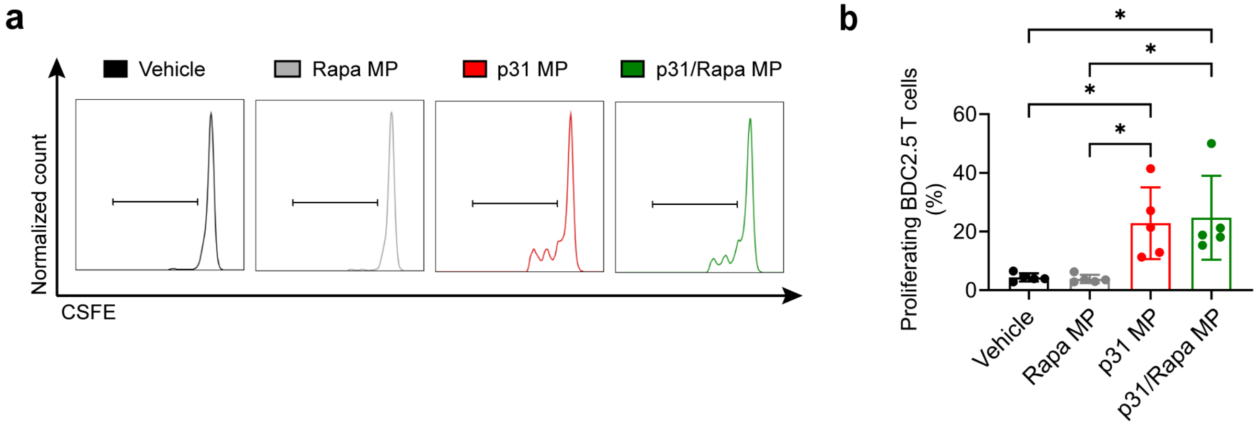

Supplemental figure 9. MPs loaded with p31 expand antigen-specific T cells in treated LNs when adoptively transferred 3 weeks after MP injection. NOD mice were injected *i.LN* with vehicle, Rapa MPs, P31 MPs or P31/Rapa MPs, and 3 weeks after treatment CSFE labeled BDC2.5 T cells were adoptively transferred into host NOD mice. 4 days after adoptive transfer, proliferation of transferred T cells in treated LNs was quantified. (a) Representative flow cytometry plots showing proliferation of BDC2.5 T cells in treated LNs by CSFE dilution. (b) Aggregate data showing proliferation of transferred T cells. N = 5 mice for all groups, and paired inguinal LNs were pooled for each mouse. The mean ± s.d. is shown. One way ANOVA with Tukey's post hoc test was used to compare each treatment within individual lymph nodes. * indicates $p < 0.05$, ** indicates $p < 0.01$, *** indicates $p < 0.001$, **** indicates $p < 0.0001$

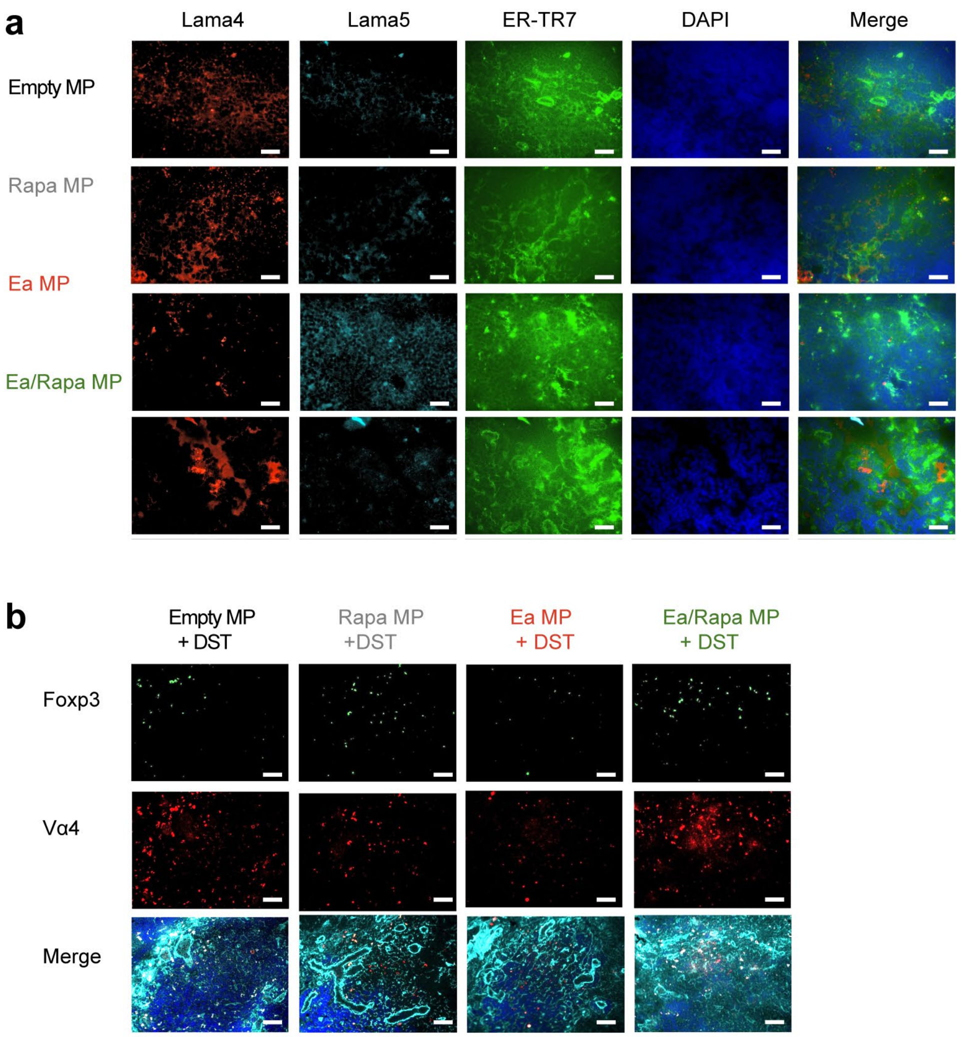

Supplementary Figure 10. Representative images including merge of all channels for data summarized in **Figure 7**. (a) corresponds to **Figure 7a-d**, **7h-k** (b) corresponds to **Figure 7g-j**.

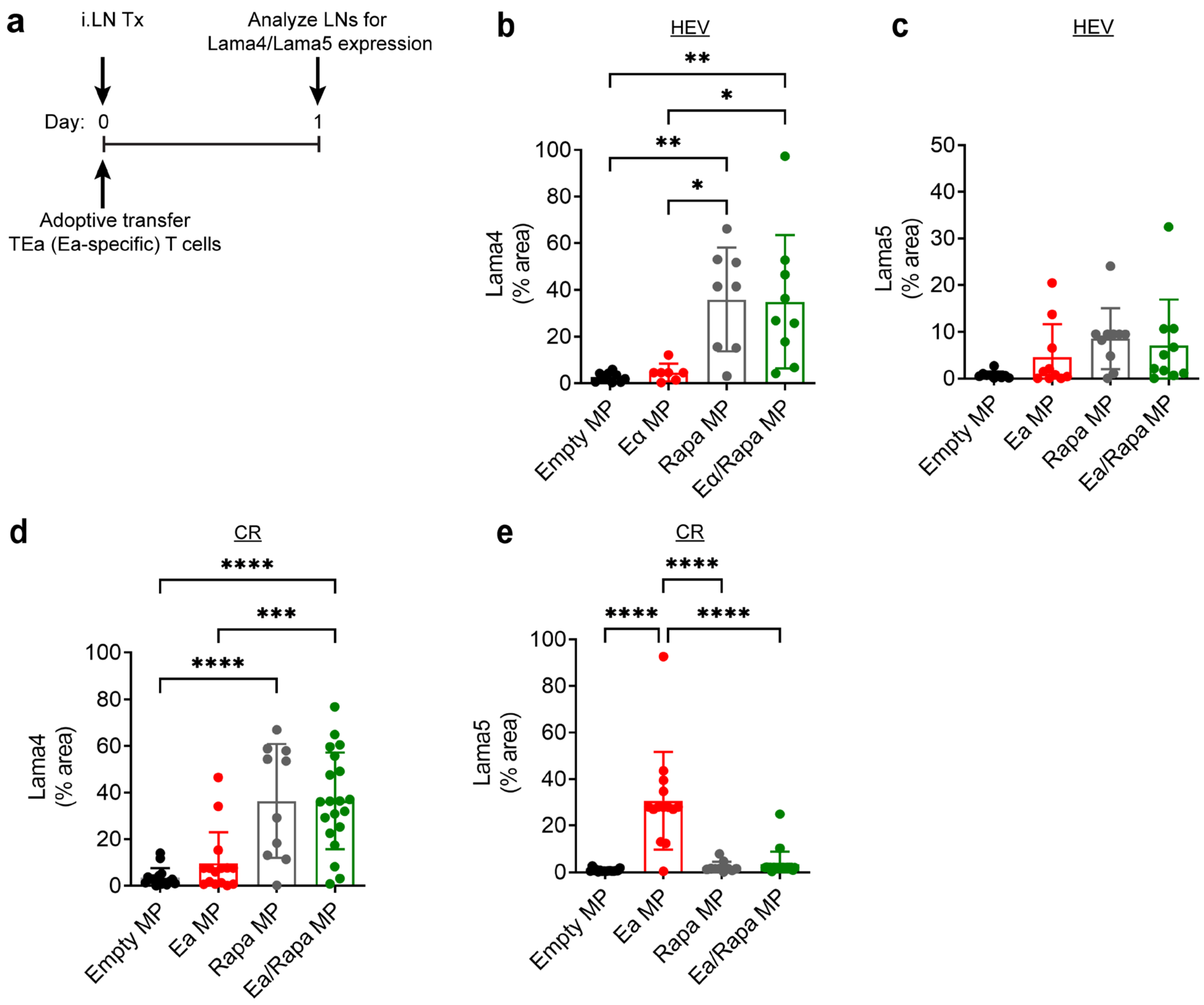

Supplementary Figure 11. (a) Schematic representation of experiment summarized in panels b-e. Percent area of laminin α4 in HEVs (c) and CRs (d) of treated LNs from mice treated with indicated groups. Percent area of laminin α5 in HEVs (e) and CRs (f) of treated LNs from mice treated with indicated groups. One way ANOVA with Tukey's post hoc test was used to compare treatment groups. Experimental details for these data are outlined in **Figure 7**. * indicates $p < 0.05$, ** indicates $p < 0.01$, *** indicates $p < 0.001$, **** indicates $p < 0.0001$

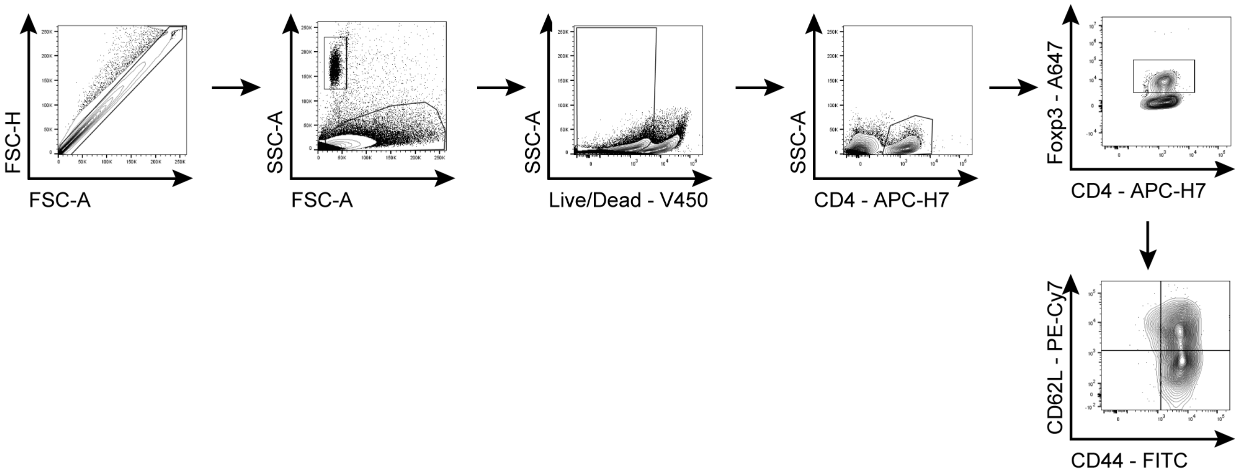

Supplementary Figure 12. Gating scheme for data summarized in **Figure 9a-d**.

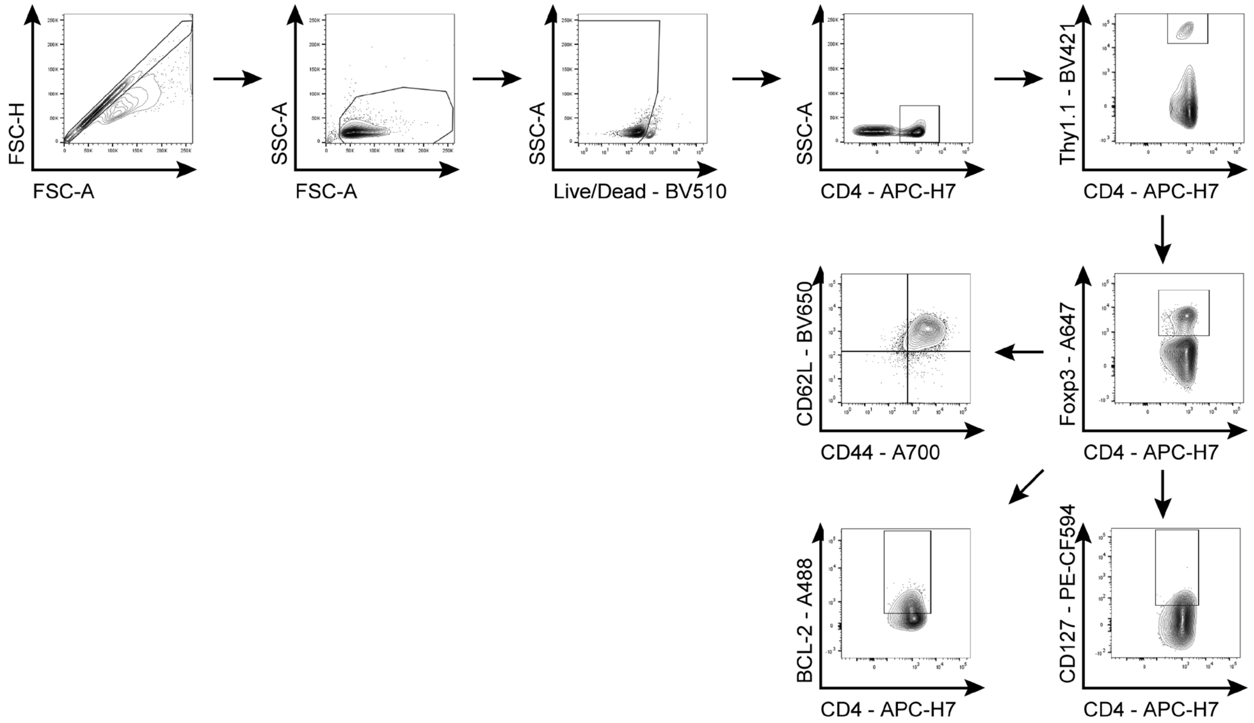

Supplementary Figure 13. Gating scheme for data summarized in **Figure 9f-k**.

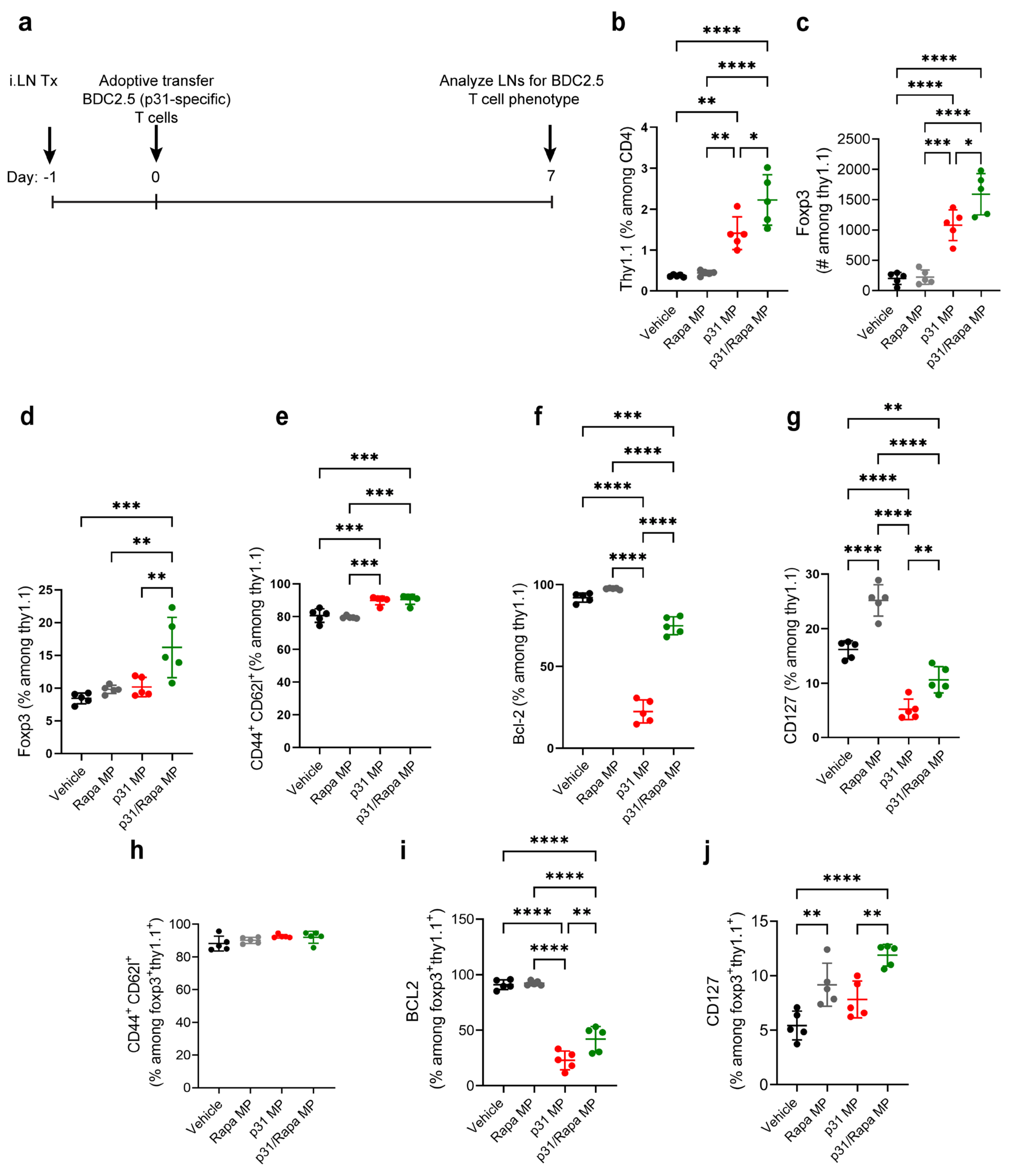

Supplementary Figure 14. Experimental details for these data are described in **Figure 9.** (a) Schematic representation of experiment summarized in panels b-j. (b) Frequency of BDC2.5-thy1.1 T cells in treated LNs. Number (c) and frequency (d) of Foxp3 expressing cells among all antigen-specific (BDC2.5-thy1.1) in treated LNs. Frequencies of (e) CD44$^+$CD62L$^+$, (f) Bcl-2, and (g) CD127 expressing cells among all antigen specific (BDC2.5-thy1.1) in treated LNs. Frequencies of (h) CD44$^+$CD62l$^+$, (i) Bcl-2, and (j) CD127

expressing antigen-specific T_{REG} in treated LNs. For panels b-j, one way ANOVA with Tukey's post hoc test was used to compare treatment groups. * indicates $p < 0.05$, ** indicates $p < 0.01$, *** indicates $p < 0.001$, **** indicates $p < 0.0001$

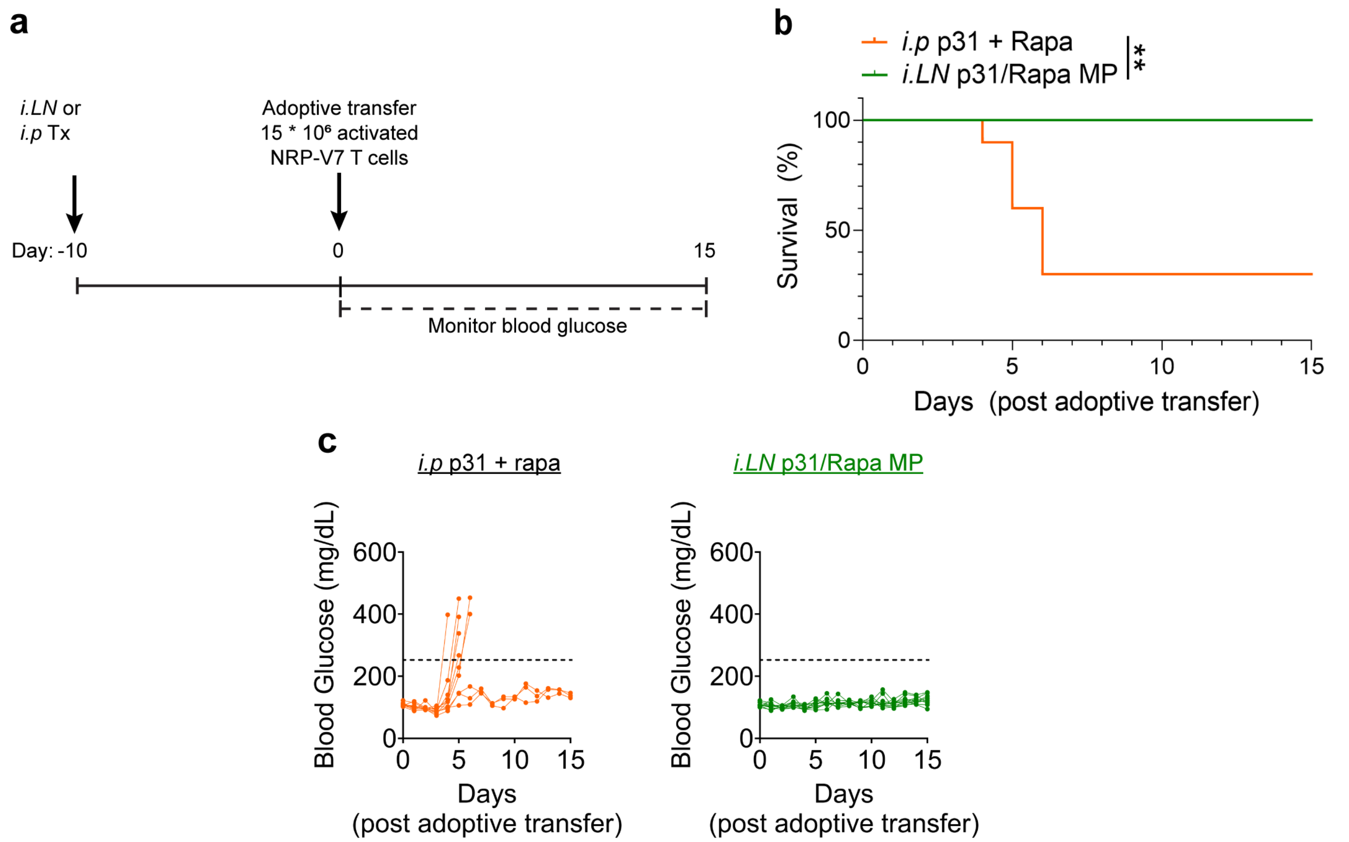

Supplemental figure 15. i.LN p31/Rapa MPs promote durable tolerance in T1D compared to soluble p31 and Rapa given *i.p.* (a) Schematic representation of NRP-V7 adoptive transfer induced T1D experiment, where host mice were treated with *i.LN* p31/Rapa MPs, or *i.p* soluble p31 with soluble Rapa 10 days before adoptive transfer of activated NOD8.3 T cells. N = 10 for each treatment group. The dose for p31 was 80µg of Rapa and 20µg of p31 for the *i.p.* treatment (b) Survival curve indicating percent of mice remaining normoglycemic. (c) Individual blood glucose traces for mice in each group. Two tailed log-rank (Mantel-Cox) was used for all pairwise comparisons between each treatment. * indicates $p < 0.05$, ** indicates $p < 0.01$, *** indicates $p < 0.001$, **** indicates $p < 0.0001$

	Diameter (μm)	p31 loading (μg peptide/mg particle)	NRP-V7 loading (μg peptide/mg particle)	Ea loading (μg peptide/mg particle)	Rapa loading (μg Rapa/mg particle)
Empty	3.7 ± 0.2	N/A	N/A	N/A	N/A
Rapa	3.6 ± 0.7	N/A	N/A	N/A	39.2 ± 6.3
p31	3.2 ± 0.2	7.4 ± 0.5	N/A	N/A	N/A
p31/Rapa	4.2 ± 0.1	8.2 ± 1.7	N/A	N/A	32.0 ± 1.9
NRP-V7	3.7 ± 0.2	N/A	3.4 ± 0.2	N/A	N/A
NRP-V7/Rapa	3.8 ± 0.5	N/A	5.2 ± 0.8	N/A	37.8 ± 4.8
Ea	3.9 ± 0.9	N/A	N/A	1.8 ± 1.0	N/A
Ea/Rapa	4.0 ± 1.3	N/A	N/A	1.2 ± 0.4	17.6 ± 2.0

Supplemental table 1. Physical properties of microparticles used throughout the studies. Particle size was measured via laser diffraction. Loading of all peptide antigens were measured by microBCA,and loading of rapamycin was measured by UV/vis spectrophotometry and are reported per mass of particles. All measurements are reported as averages of 3 or 4 batches of each particle formulation.

Statistical comparison	Summary	Adjusted P Value
late draining LN Rapa MP vs. late draining LN p31/Rapa MP	ns	>0.9999
late draining LN Rapa MP vs. treated LN Rapa MP	ns	0.1794
late draining LN Rapa MP vs. treated LN p31/Rapa MP	***	0.0006
late draining LN Rapa MP vs. early draining LN Rapa MP	ns	0.5669
late draining LN Rapa MP vs. early draining LN p31/Rapa MP	**	0.0079
late draining LN Rapa MP vs. pancreatic LN Rapa MP	ns	0.7588
late draining LN Rapa MP vs. pancreatic LN p31/Rapa MP	*	0.0179
late draining LN p31/Rapa MP vs. treated LN Rapa MP	ns	0.3406
late draining LN p31/Rapa MP vs. treated LN p31/Rapa MP	**	0.0017
late draining LN p31/Rapa MP vs. early draining LN Rapa MP	ns	0.7934
late draining LN p31/Rapa MP vs. early draining LN p31/Rapa MP	*	0.0198
late draining LN p31/Rapa MP vs. pancreatic LN Rapa MP	ns	0.9253
late draining LN p31/Rapa MP vs. pancreatic LN p31/Rapa MP	*	0.0417
treated LN Rapa MP vs. treated LN p31/Rapa MP	ns	0.1486
treated LN Rapa MP vs. early draining LN Rapa MP	ns	0.9852
treated LN Rapa MP vs. early draining LN p31/Rapa MP	ns	0.7365
treated LN Rapa MP vs. pancreatic LN Rapa MP	ns	0.9101
treated LN Rapa MP vs. pancreatic LN p31/Rapa MP	ns	0.8732
treated LN p31/Rapa MP vs. early draining LN Rapa MP	*	0.0218
treated LN p31/Rapa MP vs. early draining LN p31/Rapa MP	ns	0.9415
treated LN p31/Rapa MP vs. pancreatic LN Rapa MP	**	0.009
treated LN p31/Rapa MP vs. pancreatic LN p31/Rapa MP	ns	0.9073
early draining LN Rapa MP vs. early draining LN p31/Rapa MP	ns	0.2379
early draining LN Rapa MP vs. pancreatic LN Rapa MP	ns	>0.9999
early draining LN Rapa MP vs. pancreatic LN p31/Rapa MP	ns	0.3954
early draining LN p31/Rapa MP vs. pancreatic LN Rapa MP	ns	0.1207
early draining LN p31/Rapa MP vs. pancreatic LN p31/Rapa MP	ns	>0.9999
pancreatic LN Rapa MP vs. pancreatic LN p31/Rapa MP	ns	0.2295

Supplemental table 2. Statistical comparisons of frequencies of antigen-specific T_{REG} in mice treated with Rapa MPs and mice treated with p31/Rapa MPs in treated and all untreated lymph nodes. One way ANOVA with Tukey's post test used to compare treatment groups. This table relates to **Figure 5** in the main text.
